# Design Principles for Bispecific IgGs, Opportunities and Pitfalls of Artificial Disulfide Bonds

**DOI:** 10.3390/antib7030027

**Published:** 2018-07-28

**Authors:** Lilach Vaks, Dana Litvak-Greenfeld, Stav Dror, LeeRon Shefet-Carasso, Galia Matatov, Limor Nahary, Shiran Shapira, Rahely Hakim, Iris Alroy, Itai Benhar

**Affiliations:** 1 School of Molecular Cell Biology and Biotechnology, The George S. Wise Faculty of Life Sciences, Tel Aviv University, Tel Aviv 69978, Israel; linais07@gmail.com (L.V.); dana.litvak@gmail.com (D.L.-G.); stavdror88@gmail.com (S.D.); leeroncl@gmail.com (L.S.-C.); galiamatatov@mail.tau.ac.il (G.M.); naharyl@yahoo.com (L.N.); 2 Integrated Cancer Prevention Center, Tel Aviv Sourasky Medical Center, Sackler Faculty of Medicine, Tel Aviv University, Tel Aviv 6423906, Israel; shiranshapira@gmail.com; 3 FusiMab, Ltd., 14 Shenkar St. POB 4093 Herzelia, Israel; rahely.hakim@gmail.com (R.H.); iris.alroy@animabiotech.com (I.A.)

**Keywords:** Complementarity-determining region, Disulfide-stabilized Fv fragment, Knobs-into-holes, Monoclonal antibody, Streptavidin, Vascular endothelial growth factor

## Abstract

Bispecific antibodies (bsAbs) are antibodies with two binding sites directed at different antigens, enabling therapeutic strategies not achievable with conventional monoclonal antibodies (mAbs). Since bispecific antibodies are regarded as promising therapeutic agents, many different bispecific design modalities have been evaluated, but as many of them are small recombinant fragments, their utility could be limited. For some therapeutic applications, full-size IgGs may be the optimal format. Two challenges should be met to make bispecific IgGs; one is that each heavy chain will only pair with the heavy chain of the second specificity and that homodimerization be prevented. The second is that each heavy chain will only pair with the light chain of its own specificity and not with the light chain of the second specificity. The first solution to the first criterion (knobs into holes, KIH) was presented in 1996 by Paul Carter’s group from Genentech. Additional solutions were presented later on. However, until recently, out of >120 published bsAb formats, only a handful of solutions for the second criterion that make it possible to produce a bispecific IgG by a single expressing cell were suggested. We present a solution for the second challenge—correct pairing of heavy and light chains of bispecific IgGs; an engineered (artificial) disulfide bond between the antibodies’ variable domains that asymmetrically replaces the natural disulfide bond between CH1 and CL. We name antibodies produced according to this design “BIClonals”. Bispecific IgGs where the artificial disulfide bond is placed in the CH1-CL interface are also presented. Briefly, we found that an artificial disulfide bond between V_H_ position 44 to V_L_ position 100 provides for effective and correct H–L chain pairing while also preventing the formation of wrong H–L chain pairs. When the artificial disulfide bond links the CH1 with the CL domain, effective H–L chain pairing also occurs, but in some cases, wrong H–L pairing is not totally prevented. We conclude that H–L chain pairing seems to be driven by V_H_–V_L_ interfacial interactions that differ between different antibodies, hence, there is no single optimal solution for effective and precise assembly of bispecific IgGs, making it necessary to carefully evaluate the optimal solution for each new antibody.

## 1. Introduction

Therapeutic monoclonal antibodies (mAbs) are the leading class of biologics that offer exciting opportunities to the biomedical and biotechnological communities [1]. Bispecific antibodies (bsAbs) are a class of antibodies that have two different antigen binding sites [2,3]. As such they offer unique opportunities that may overcome some limitations of existing therapeutic mAbs such as co-clustering of cell-surface receptors or targeting immune effector cells to kill cancer cells [4].

There are many molecular designs of bsAbs, the number of formats now exceeds 120 [5]. Many of the bsAb designs involve linking small monospecific antibody fragments in tandem. Although such small fragments are currently leading the clinical development of bsAbs, they have some limitations (that are inherent for small antibody fragments) in stability, solubility and pharmacokinetic properties [2,6]. Thus, it is expected that bsAbs of the IgG format will increasingly become more common [7,8,9].

Existing approaches for producing native IgG-like bsAbs also have limitations. Some solutions involve using two different heavy chains with a common light chain [10]. Other solutions involve assembling half antibodies in vitro to be combined later to an IgG format. Other solutions involve extensive engineering of the Fab arm interface [11], or require non-natural crossing over of heavy and light chains [9], potentially leading to concerns about ease of development and immunogenicity.

To efficiently produce a bsAb in a native IgG format, two challenges should be met; one is that each heavy (H) chain will only pair with the heavy chain of the other specificity (H–H heterodimerization) and that homodimerization will be prevented. The second is that in the Fab arm interface, each heavy chain will only pair with its cognate light chain and will not pair with the light chain of the other specificity.

Here we present a solution for the efficient engineering of the Fab arm interface of bispecific IgGs. Our solution involves eliminating in one Fab arm the native disulfide bond between the heavy and light chain and replacing it with an artificial disulfide bond between cysteines that are located at interfacial positions of the V_H_ and V_L_ domains. We further show that the cysteines introduced into the variable domains, not only provide for artificial disulfide bonding of the H and L chains but also prevent wrong chain pairing (between WT H chain to engineered L chain and vice-versa), thus facilitating correct arrangement of the Fab arm interface of the bsAb. Our bsAbs are presented in the context of knobs-into-holes (KIH) as a solution for heavy chain heterodimerization. KIH was the first molecular design for promoting heavy-chain heterodimerization of bsAbs in IgG format [10,12]. It involves asymmetrically mutating interfacial residues in the CH3 domains of the two parental mAbs so the knob (T366W) and hole (T366S, L368A, and Y407V) mutations allow H–H heterodimerization, while Knob-Knob association is prevented by steric repulsion while hole-hole homodimers are severely destabilized. KIH is regarded as the “gold standard” among molecular designs for H–H heterodimerization [13]. Although we chose KIH, since the H–H and H–L pairing are orthogonal, our H–L pairing solution can be combined with other approaches for H–H heterodimerization.

Further, we describe the production and evaluation of bsAbs where the artificial disulfide bond is formed between cysteine residues inserted into the CH1 and CL domains. Some of these bsAbs are similar in design to bsAbs that were developed by Medimmune [14]. However, when tested with our model antibodies, while bsAbs were efficiently assembled, incorrect H–L chain pairing was not completely prevented. We suggest that our “BIClonals” design (based on the artificial disulfide bond between the V_H_ and V_L_ domains), presented here for several bsAbs, (with murine, humanized and human variable domains) produced by expression in *E. coli* and in mammalian cells culture, applies to kappa (κ) and to lambda (λ) light chain pairing with heavy chains, minimally deviates from a native IgG format as it involves only four point mutations in the Fab arm interface of one side of the bispecific IgG.

## 2. Materials and Methods

### 2.1. Construction of Expression Vectors

Antibody genes were prepared by total gene synthesis optimized for expression in the particular expression host (*E. coli* or mammalian cells). In some cases, antibody variable domains-coding DNA was amplified by the polymerase chain reaction (PCR) from existing plasmid vectors. The antibody genes were cloned into separate expression vectors enabling either cytoplasmic expression in *E. coli* or secretory expression by HEK293 cells or by CHO cells. Amino acid residues of antibody chains are numbered according to the Kabat numbering scheme for the variable domains and constant domains of light chains [15] and the European numbering scheme of the constant regions of heavy chains [16]. The bacterial expression vectors were based on the pHAK plasmids described in [17]. The mammalian expression vectors were based on the pcDNA3.4, cytomegalovirus (CMV) promoter-controlled plasmid provided as part of the Expi293™ system or proprietary retroviral vectors that were used by the contract research organization (CRO) Catalent to prepare bsAbs in CHO cells. Point mutations were introduced into antibody constant domains or variable domains by overlap-extension PCR essentially as described [18]. 

### 2.2. Production of Antibodies in E. coli

Some of the antibodies that were produced during the study were made by bacterial expression using the Inclonals method for production of full-length IgGs in *E. coli* bacteria [17]. Further details are provided in the supplement (Method S1).

### 2.3. Transient Antibody Expression in HEK293 Cells

Transient expression of IgGs was carried out using the Expi293™ system (Thermo Fisher, Waltham, MA, USA) according to protocols provided by the supplier. Cells were transfected with two plasmids for mAb production of with four plasmids for bsAb production, using a 1:2 heavy-chain: light chain plasmid DNA ratio. IgGs were purified from conditioned media harvested 4–6 days post-transfection. The conditioned medium was filtered using a 0.45 µm filter and the IgG was purified by protein-A (or MabSelect, GE Healthcare, Chicago, IL, USA) affinity chromatography or by sequential Kappa-select–Lambda-select (GE Healthcare, Pittsburgh, PA, USA) affinity chromatography according to the supplier’s recommendations. Purified IgGs were stored in Phosphate-buffered saline (PBS) at −80 °C.

### 2.4. Stable Antibody Expression in CHO Cells

This was carried out using a pseudo typed, high-titer retroviral vector which generated stable transduced mammalian cells (see Supplement, Method S2).

### 2.5. SDS-PAGE Electrophoresis, Immunoblotting and ELISA

Were carried out as described in the supplement (Methods S3, S4, S5).

### 2.6. Surface Plasmon Resonance Analysis

Experiments for evaluation of antigen binding kinetics and determination of affinity constant of the antibodies were performed using the Biacore T200 instrument [19] as described in the supplement (Method S6).

### 2.7. Thermal Stability Assay

Thermal unfolding experiments are highly appreciated methods to quantify protein stability. The thermal stability of a given protein is typically described by the thermal unfolding transition midpoint Tm (°C), at which half of the protein population is unfolded. In this study we used a NanoDSF assay for determination of the Tm values of each of the investigated antibodies. NanoDSF (DSF = Differential Scanning Fluorimetry) measurements were performed using the Prometheus NT.48 instrument by NanoTemper Technologies GmbH (Munich, Germany) [20] as described in the supplement (Method S7). 

### 2.8. MTT Cell Viability Assay

Was carried out as described in the supplement (Method S8).

### 2.9. Animal Studies

Animal studies were carried out by certified service providers. The blood pharmacokinetics study was carried out by Science in Action Biotech (Israel) and the xenograft study by Charles River (Wilmington, MA, USA). All experiments were approved by the local governments. Further details are provided in the supplement (Method S9 and S10).

### 2.10. Statistical Analysis

All raw data were processed using statistics software Prism (GraphPad, La Jolla, CA, USA) for Windows 3.03.

## 3. Results

### 3.1. Design of Artificial Disulfide Bonds for H–L Chain Pairing

**Figure 1:**
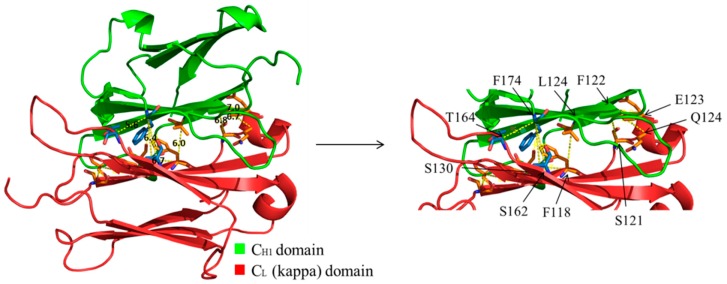
C_H1_-C_L_ interface with the potential positions for a stabilizing disulfide bond formation. C_H1_ and C_L_ domains (of Light chain kappa chain) are presented colored green and red, respectively, with the potential positions for disulfide bond formation at the C_H1_-C_L_ interface.

In this study we were inspired by studies from the 1990s where artificial disulfide bonds, linking antibody V_H_ to V_L_ domain were used to stabilize *E. coli*-expressed Fvs, namely, making disulfide-stabilized Fvs (dsFvs) [21,22,23]. As the positions for placing the cysteines were known from these studies, we did not have to design them de-novo. In contrast, when we initiated the study, positions for placing cysteines for the formation of artificial disulfide bonds between the CH1 and CL domains were not known. Therefore, for identifying the most suitable positions within the CH1-CL interface that, upon mutagenesis to cysteines could create stabilizing interaction between the H and the L chains and thus be used for a correct dimerization of the two chains, we used 3D structural models of human IgGs. We performed structural analysis of this interface using the molecular visualization system PyMol. Two main criteria were taken into consideration: First, the distance between the α-carbon of the two potential residues (Cα–Cα distance) should be in the range of 5.0–7.6 Å to enable disulfide bond formation and allow for possible movement of the main chain [10,21]. Second, the side chains should face each other. The search resulted in ten different pairs of positions in which a disulfide bond could theoretically be introduced as an optional stabilizing interaction. After conducting structural alignments of both CH1 and CL domains in existing structures, (Protein Data bank (PDB) files: 1UCB, 2ZKH and 3DVG) we chose seven of the mutant pairs to produce and analyze. A view of the interface and the examined positions are displayed in Figure 1 and the position pairs are listed in Table 1.

In our model molecules, one Fab arm was engineered while the second remained unmodified. Since our focus was on studying H–L pairing, as a solution for H–H heterodimerization we chose the well-benchmarked “Knobs-into-Holes” (KIH) solution for the two heavy chains heterodimerization [10].

### 3.2. Evaluation of H–H Chain Heterodimerization in Refolded Bispecific IgGs

**Figure 2:**
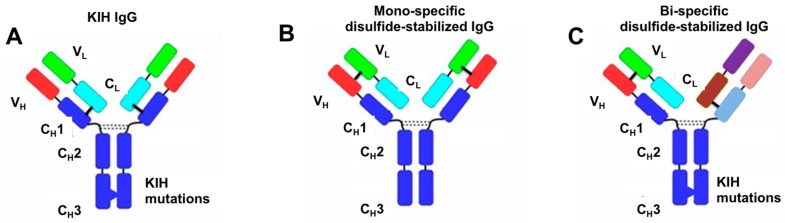
Schematic representation of our work strategy for production of mono- and bi-specific disulfide stabilized antibodies. (**A**) Scheme of an IgG antibody produced by the “knobs-into-holes” (KIH) approach (10), there are two different heavy chains but a common light chain; (**B**) Scheme of a mono-specific disulfide stabilized antibody (also named “disulfide-stabilized IgG”) which contains engineered disulfide bonds between antibody V_H_ and V_L_ domains symmetrically engineered in both Fab arms, with the native disulfide bonds connecting C_L_ and C_H_1 eliminated; (**C**) Scheme of a bi-specific antibody prepared according to our “BIClonals” approach. There are two different heavy chains, each paired to its cognate light chain. The KIH mutations correspond to T366W (“knob”), T366S, L368A, Y407V (“hole”), S354C and Y349C (cysteine replacement mutations at CH3 region of “knob” and “hole”, respectively). Fab arm interface disulfide-stabilizing mutations correspond to V_H_ A44C and V_H_ C222A, V_κ_ A100C and C_κ_ C214del, V-λ A100C and Cλ C214A (or C214G that was used in the case of the anti streptavidin (SA) antibody).

When we initiated the study, KIH was demonstrated only with bsAbs produced in mammalian cells culture. Since we chose to evaluate chain pairing using refolded IgGs from bacterial expression, we initially studied if KIH is suitable for H–H heterodimerization for refolded IgGs. We studied our design principles by producing bispecific IgGs by refolding *E. coli*-produced H and L chains according to the “Inclonals” technology [17] (see method S1). The Inclonals technology was developed for producing IgGs and IgG based fusion proteins by refolding a heavy chain mixed with the corresponding light chain. For producing bsAbs, it was modified to involve refolding two different heavy chains mixed with two different light chains. The model antibodies we used for these proof-of concept experiments were the anti ErbB2 antibody FRP5 [24] and the anti CD30 antibody T427 [25]. A scheme of the molecular design of antibodies that we tested throughout this study is shown in Figure 2.

**Figure 3:**
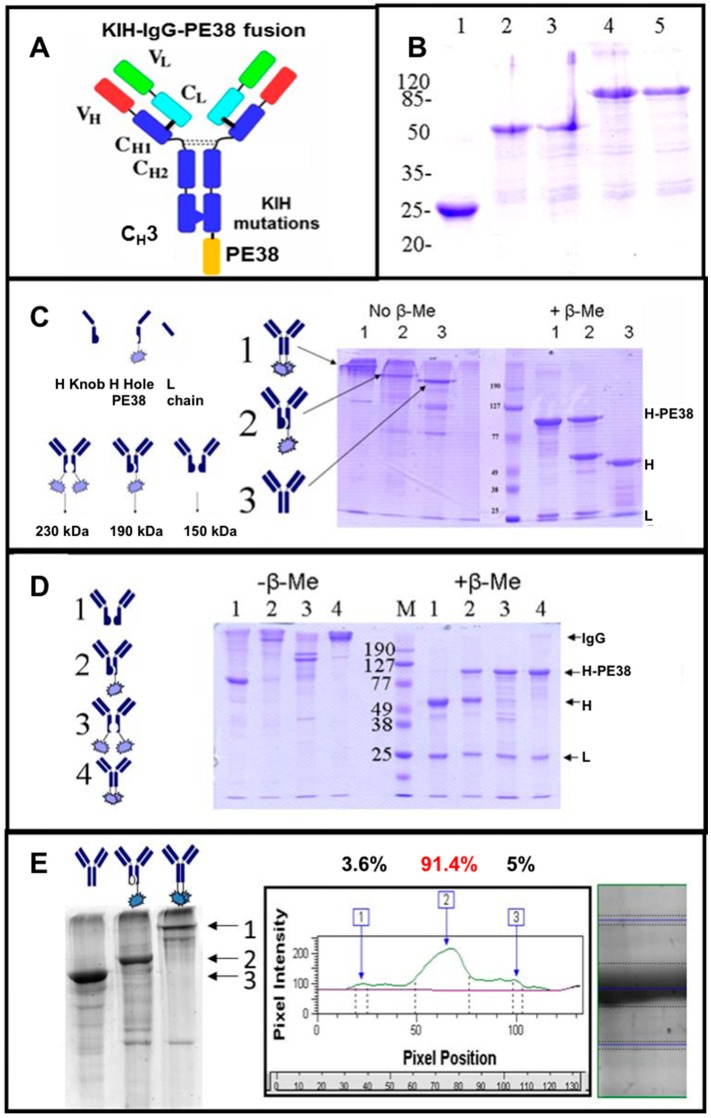
Evaluation of refolded IgGs carrying KIH mutations. (**A**) Scheme of an IgG antibody carrying KIH mutations with a truncated fragment of Pseudomonas exotoxin (PE38) fused to the WT heavy chain or to the “hole” heavy chain; (**B**) Purified inclusion bodies of the heavy and light chains dissolved in 6M guanidinium hydrochloride buffer: (1) T427 Light chain; (2) T427 Heavy chain; (3) T427-Heavy chain-knob; (4) T427-Heavy chain-PE38; (5) T427-Heavy chain-hole-PE38; (**C**) Determination of heavy-heavy chains heterodimerization (1) WT IgG with PE38 fused to both its heavy chains; (2) KIH IgG in which a “hole” heavy chain is fused to PE38 toxin fragment; (3) WT IgG; (**D**) Heavy-heavy chains homo-versus heterodimers formation. (1) “Knob-knob” version; (2) KIH IgG in which a “hole” heavy chain is fused to PE38 toxin fragment; (3) “Hole-hole” version; (4) IgG displaying PE38 fused to its heavy chains; (**E**) Protein band density analysis for determination of heavy chains homo-versus heterodimers formation. By 1, 2 and 3 we marked the expected positions of the examined antibodies (1) WT IgG; (2) KIH IgG in which a “hole” heavy chain is fused to PE38 toxin fragment; (3) IgG with PE38 fused to both heavy chains.

**Figure 4:**
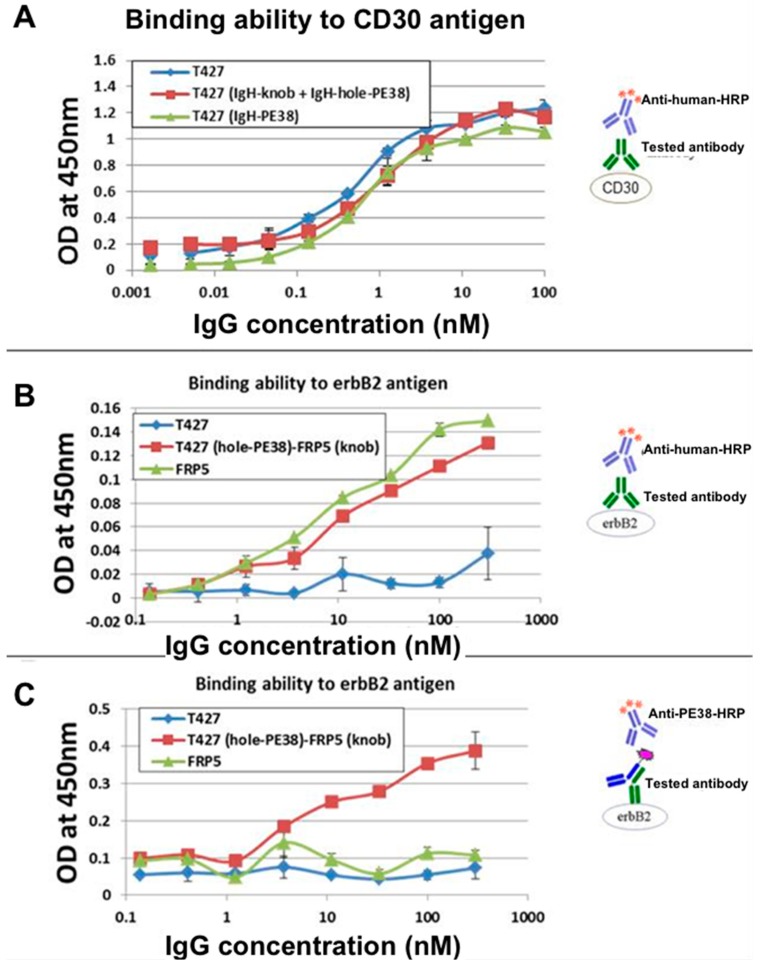
Evaluation of antigen binding by refolded IgGs and IgG-KIH derivatives. (**A**) Evaluation by ELISA of CD30 binding by T427 IgG and T427-PE38 (PE38 fused to each heavy chain) in comparison to the KIH version of T427; (**B**) Evaluation by ELISA of ErbB2 binding by KIH FRP5-T427-PE38 (Light chain-FRP5 + Light chain-T427 + Heavy chain-FRP5-knob+Heavy chain-T427-hole-PE38) compared to the parental IgGs; (**C**) Evaluation by ELISA of heavy chains heterodimer formation. The KIH T427-FRP5 PE38 IgG bound ErbB2 (binding by the FRP5-knob heavy chain) and its T427-hole-PE38 was identified by anti-PE38 antibodies.

To distinguish between H–H homodimers to heterodimers we expressed the “hole” heavy chain as a fusion protein with a truncated part of Pseudomonas exotoxin (PE38, see [17]). To allow comparison to a monoclonal IgG, model antibodies were anti CD30 T427 antibody produced as an unmodified mAb, as a mAb fused to PE38 or as a KIH engineered mAb with one knob and one hole heavy chains (note: this is NOT a bsAb). The two heavy chains and the common light chain were produced as inclusion bodies upon expression in *E. coli*. They were purified, reduced, mixed, refolded and purified according to the Inclonals protocol. Samples of the purified antibodies were analyzed by sodium dodecyl sulfate polyacrylamide gel electrophoresis (SDS/PAGE). As shown in Figure 3, the molecular weight of the heavy chain-PE38 fusion protein was ~90 kDa, so, a heavy chain homodimer (~100 kDa) and a heavy chain-PE38 homodimer (~180 kDa), thus providing the clear differentiation between homo- and heterodimer IgGs according their molecular weights (as a fully assembled IgG, the “knob-hole” heterodimer appears as 190 kDa molecule, the “knob-knob” homodimer and the “hole-hole” homodimer appear as 150 kDa and 230 kDa, respectively) (Figure 3C). The SDS/PAGE analysis clearly shows that the unmodified mAb was produced according to the expected molecular weight (MW) of a fully assembled IgG and so was the KIH engineered IgG (Figure 3C). The detailed homo-versus heterodimer analysis demonstrated more than 90% heterodimers formation that was an outstanding result for in vitro refolded molecules (Figure 3E). Furthermore, an attempt to produce homodimer versions of IgG by refolding only one heavy chain type (either “knob” or “hole”) failed to produce fully-assembled IgG and resulted in the accumulation of partially-assembled antibodies (Figure 3D lanes 1 and 3) while the KIH and unmodified IgGs were assembled correctly (Figure 3D lanes 2 and 4). In addition, the produced IgG KIH molecules preserved their antigen binding ability in comparison to unmodified IgGs and IgG-PE38 fusion protein, as evident from their analysis by ELISA (Figure 4A).

To evaluate the production of KIH-based bispecific molecules the KIH T427-FRP5 antibody was constructed. This IgG consisted of 4 different chains: FRP5-knob and T427-hole-PE38 heavy chains, and FRP5 wild-type (WT, unmodified sequence) and T427 WT light chains. Note that for these KIH bsAbs, H–L chain pairs still assemble at random and the solution for correct H–L pairing will be described in the next section. The PE38 toxin in this construct was used as a tag to detect the T427 heavy chain. The mono-specific T427 and FRP5 IgGs served as controls. SDS/PAGE analysis of the refolded bsAb revealed efficient assembly of correct heavy chain heterodimers (not shown). Using ELISA, we demonstrated the antibodies’ binding ability to each one of their respective antigens. The sandwich ELISA analysis (Figure 4C) evaluated the antibody binding to ErbB2 (the antigen of the FRP5 arm) while the T427-PE38 chain was detected using anti PE antibodies. This assay demonstrated the presence of T427-FRP5 heavy chains heterodimer that was able to bind both antigens. These results suggest that the “knobs-into-holes” approach provides a solution for heavy chains heterodimerization for refolded bi-specific IgGs and that the Inclonals method can be implemented for studying chain assembly of bispecific IgGs. 

### 3.3. Evaluation of H–L Chain Heterodimerization in Refolded IgGs

**Figure 5:**
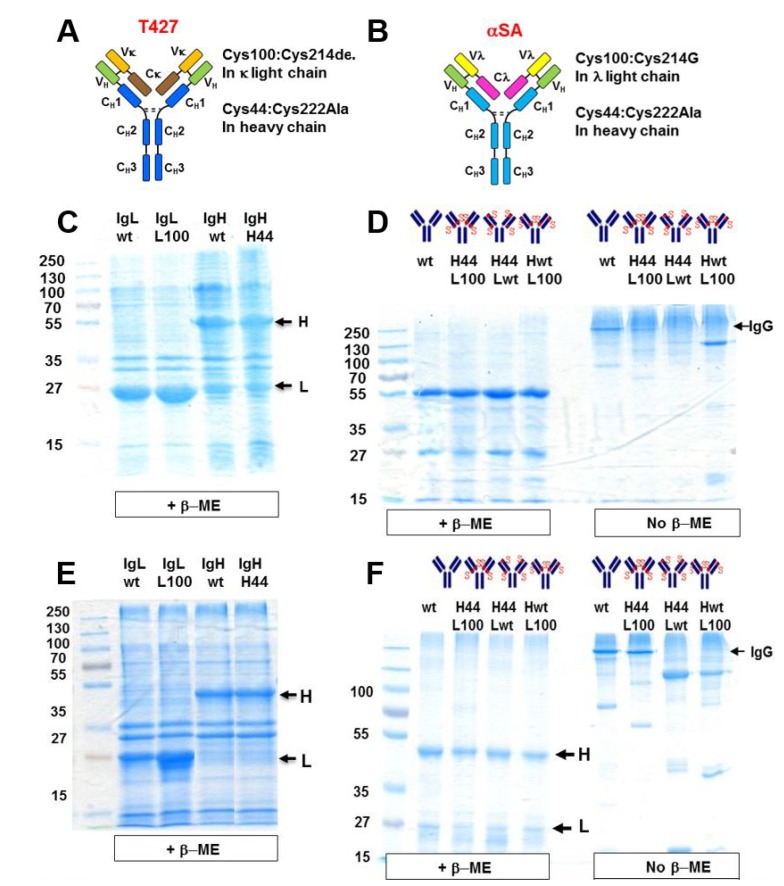
Analysis of heavy-light (κ and λ) chains pairing stabilized or not-stabilized by artificial inter-chain disulfide bonds. (**A**) Schematic structure of the “disulfide-stabilized IgGs” (T427) antibody (that has a kappa light chain); (**B**) Schematic structure of “disulfide-stabilized IgGs” anti SA (αSA) antibody (that has a lambda light chain); (**C**) T427 heavy and light chains inclusion bodies solubilized in 6M guanidinium hydrochloride analyzed by 12% SDS/PAGE under reducing conditions; (**D**) Evaluation by SDS/PAGE (12% gel, left gel under reducing and right gel under non-reducing conditions) of heavy-κ light chains pairing. WT and H44-L100 refer to IgGs in which an inter-chain disulfide bond can be formed either by the native disulfide bond connecting C_L_–C_H_1 domains (WT) or V_H_–V_L_ domains (H44-L100)). H44-L WT and H WT-L100 represent the molecules in which an inter-chain disulfide bond cannot be formed; (**E**) Anti SA heavy and light chains inclusion bodies solubilized in 6M guanidinium hydrochloride; (**F**) Evaluation by SDS/PAGE (12% gel, left gel under reducing and right gel under non-reducing conditions) of heavy-λ light chain pairing. WT and H44-L100 refer to IgGs in which inter-chain disulfide bond can be formed (either connecting C_L_–C_H_1 domains (WT) or V_H_–V_L_ domains (H44-L100)). H44-L WT and H WT-L100 represent the molecules in which inter-chain disulfide bond cannot be formed.

As stated in the introduction, the requirement for an effective solution of the Fab arm pairing of a bispecific IgG is that every heavy chain will only pair with its cognate light chain and that wrong pairing will be prevented. To evaluate how H–L pairing is affected by our design principles, we constructed symmetrically engineered derivatives of the anti CD30 antibody T427 and of an anti-streptavidin IgG (anti-SA, also named αSA) (αSA was described in [26], it is a human antibody that was isolated as a scFv with a lambda V_L_ from a human scFv phage display library [27] and converted to a human IgG1 for this study). SDS/PAGE (Figure 5) shows the analysis of the assembly of these two antibodies as “correct” pairs (when both the H and L chains are unmodified or when they are both engineered at the H–L interface (Figure 5D,F, wt and H44/L100, respectively, separated under non-reducing conditions)) or “wrong” pairs (one chain unmodified and the other modified, (Figure 5D,F, H44/Lwt and Hwt/L100 separated under non-reducing conditions)).

As shown, only when correct pairs of chains were refolded together were fully assembled IgGs obtained, which was true for T427 (murine V_H_ and V_L_ domains with a κ light chain, Figure 5D) and anti SA (human V_H_ and V_L_ domains with a λ light chain, Figure 5F).

**Figure 6:**
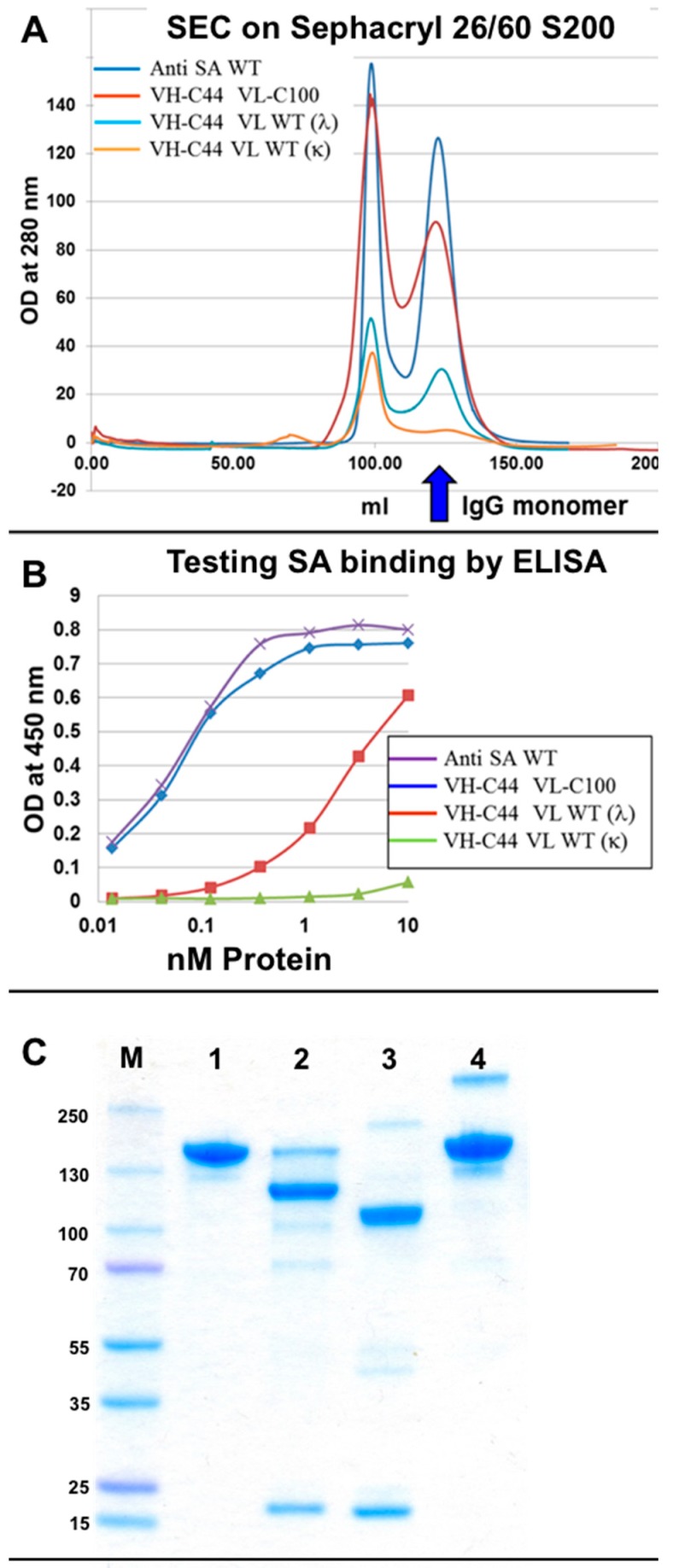
Evaluation of “wrong” H–L pair formation. Four antibodies were evaluated: a WT αSA, a V_H_ C44 heavy chain of αSA refolded with V_L_ C100 λ light chain of αSA, a V_H_ C44 heavy chain of αSA refolded with a WT λ light chain of αSA, a V_H_ C44 heavy chain of αSA refolded with WT κ light chain of T427 The four antibodies were refolded at the same scale, purified over a MabSelect column and the column eluate was loaded on a Sephacryl 26/60 S200 size exclusion chromatography (SEC) column (**A**). (**B**) Evaluation of SA binding by the monomeric fractions from (**A**) by ELISA. (**C**) antibodies expressed in Expi293™ mammalian cells: (1) The commercial therapeutic mAb Infliximab shown as a reference to full-size IgG; (2) αSA Heavy chain hole T427 Heavy chain knob C44 C222A, with T427 Light chain WT; (3) αSA Heavy chain WT T427 Light chain C100 C214Δ; (4) αSA Heavy chain WT, T427 Light chain WT.

To evaluate more thoroughly if “wrong” H–L pair formation is permitted, we further evaluated the formation and antigen binding properties of the αSA IgG. With this antibody, antigen binding is contributed by both H and L chains and when the light chain is absent or replaced, antigen binding is severely compromised (see Figure S1). Moreover, we also found that the αSA IgG has a very stable H–L Fab arm interface and in fact assembles correctly and binds antigen as well as the WT αSA IgG even in the absence of an inter-chain disulfide bond. In the experiments described in Figure 6, we evaluated the efficiency of assembly and the antigen binding properties of four antibodies. One was the unmodified (WT) αSA IgG, the second was an IgG comprising a mutated heavy chain (with V_H_ A44C and C_H_1 C222A mutations) combined with a mutated light chain (with V_λ_ A100C and C_λ_ C214G (C214G was used to make the αSA antibody, for other antibodies with lambda light chains we use C214A)), the third was an IgG comprising a mutated heavy chain combined with the unmodified (WT) λ light chain of αSA and the fourth was an IgG comprising a mutated heavy chain combined with an unmodified (WT) κ light chain of the anti CD30 antibody T427. All the 4 antibodies had an unmodified (not KIH) Fc of human gamma 1 isotype (see Figure 5A). The efficiency of assembly was determined by the quantity of antibody eluting from the MabSelect column, as evident from the height of the monomer peak eluting from the size-exclusion chromatography (SEC) column while antigen binding was evaluated by ELISA. The results are shown in Figure 6.

As shown, the fully modified and the WT αSA IgG were produced with an equal efficiency (Figure 6A) and bound SA in ELISA with similar avidity (Figure 6B). In contrast, the assembly of IgGs that combines a Cys44 modified heavy chain with an unmodified κ or λ light chain was severely impaired (Figure 6A) (which is in agreement with the results shown in Figure 5) as was their antigen binding ability (Figure 6B). Similar results were obtained when an antibody combining a mutated H chain with a WT Kappa chain was expressed in Expi293™ mammalian cells (Figure 6C). The reciprocal experiment, where a WT H chain was combined with a mutated (C100) L chain was carried out only in Expi293™ mammalian cells. This molecule completely failed to assemble as a full-size IgG. These results suggest that not only do the mutations that facilitate artificial V_H_–V_L_ disulfide bond formation allow the pairing of the engineered chain, but they actively inhibit the formation of wrong H–L chain pairs.

### 3.4. Production of bsAbs by Refolding and Their Functional Evaluation

**Figure 7:**
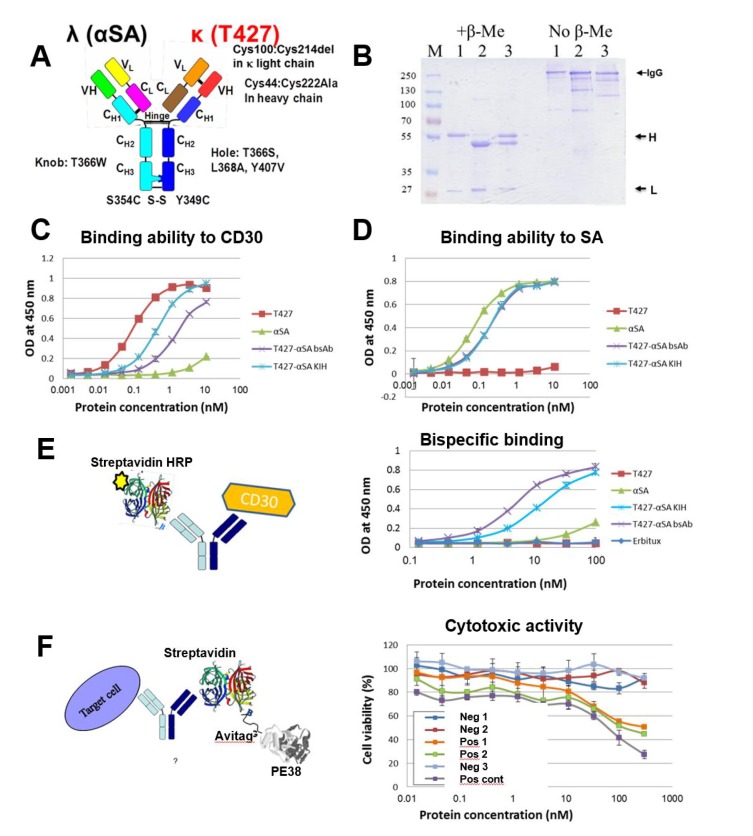
Characterization of refolded T427 (disulfide stabilized)-αSA bi-specific antibody. (**A**) The schematic structure of T427 (disulfide stabilized)-αSA bi-specific antibody. In this model, the T427 antibody (that has a κ light chain) arm was modified to carry disulfide stabilizing mutations (V_H_ C44 and V_L_ C100) while the αSA antibody (that has a λ light chain) arm remained unmodified at the Fab arm interface; (**B**) Sodium dodecyl sulfate polyacrylamide gel electrophoresis (SDS/PAGE) analysis (10% gel) of protein A purified T427 WT (1), αSA WT (2) and T427(disulfide stabilized)-αSA bsAb (3) antibodies; (**C**) Evaluation by ELISA of CD30 binding by the T427 (disulfide stabilized)-anti SA in comparison with WT and KIH (stabilized by KIH at the C_H_3-C_H_3 interface but unmodified at the Fab arm interface) T427 IgGs; (**D**) Evaluation by ELISA of SA binding by the T427 (disulfide stabilized)-αSA antibody in comparison with WT and KIH IgGs; (**E**) Evaluation by ELISA of simultaneous binding of both CD30 and SA by the T427 (disulfide stabilized)-αSA bsAb. The CD30 antigen was used to coat an ELISA plate, incubated with the evaluated antibodies and binding was detected using horseradish peroxidase (HRP) conjugated streptavidin; (**F**) Evaluation of T427 κ (disulfide stabilized)-αSA bispecificity by recruiting an effector function (cytotoxic activity) to target cells. **Neg 1**: T427 mAb mixed with αSA mAb and with SA and biotinylated Avitag-PE38; **Neg 2**: αSA mAb and with SA and biotinylated Avitag-PE38; **Pos 1**:T427/αSA bsAb with SA and biotinylated Avitag-PE38; **Pos 2**: T427/αSA bsAB (KIH only) with SA and biotinylated Avitag-PE38; **Neg 3**: SA and biotinylated Avitag-PE38 (no antibody present); **Pos cont**: A T427 IgG-PE38 fusion protein as positive control. Cytotoxicity was evaluated using an MTT assay. The evaluated antibodies were mixed with a streptavidin-PE38 complex and added to A431/CD30 cell line. Streptavidin-PE38 complex served as a control for non-specific toxic effect. T427-di-PE38 immunotoxin [17] served as a positive control.

After studying chain pairing using “disulfide-stabilized IgGs”, we turned to study real bsAbs. The bi-specific T427-αSA antibodies were produced as follows: either the T427 or the αSA arm had an engineered Fab arm while the other was not modified at the H–L Fab arm interface. The heterodimerization of the Fc region was provided by the introduction of KIH mutations (Figure 2C). The evaluation of binding properties of the obtained bi-specific molecules confirmed an up to 10-fold decrease in independent binding of each antigen that can be explained by the mono-valent nature of antigen binding of a bsAb vs. the reference bivalent mAb (Figure 7C,D). The successful binding of two antigens simultaneously was also demonstrated (Figure 7E).

As an additional proof of bi-specificity we evaluated the cytotoxic activity of a toxin recruited to target cells by the bsAb. The anti-CD30 arm of the T427-anti SA bsAb was responsible for recognition of the A431/CD30 cell line [28] while the anti-SA arm recruited the streptavidin-biotinylated PE38 toxin complex (prepared as described in the methods S11 and S12). As shown in Figure 7F, cell killing could be observed when the cells were treated with the bsAb-SA-PE38 complex (a T427-anti-SA bsAb where each H chain is correctly connected to its cognate L chain) and by the KIH-only bsAb-SA-PE38 complex (a T427-anti-SA bsAb where the H chains associate with the L chains at random, which is why it is less potent then the fully-engineered bsAb). In all cases, when cytotoxic activity can be observed it was because the biotinylated Avitag-PE38 toxin was recruited by the anti-SA arm via a biotin-streptavidin complex). The negative controls that included mixing T427 mAbs with anti-SA mAb, anti SA mAb alone, SA + biotinylated Avitag-PE38 did not exhibit any cells killing. Similar results were obtained when the binding and cytotoxicity evaluation was also carried out on a “reciprocal” bsAb, namely one with the T427 Fab arm unmodified and the anti SA Fab arm engineered (not shown). These results, proving the ability of a “disulfide stabilized” bsAb to recruit an effector molecule further support the bispecific characteristics of the evaluated antibodies.

### 3.5. Evaluation of Additional Positions for Disulfide-Stabilization of the Fab Arm Interface of bsAbs

We chose the pair of mutations V_H_ C44 V_L_ C 100 based on reports of this being the preferred combination in context of dsFvs [29]. However, a number of possible positions for artificial disulfide bonds that might stabilize the Fv fragments of antibodies had been identified and evaluated in the dsFv format. To evaluate the potential of additional cysteine pairs to facilitate disulfide stabilization of the Fab arm interface in the context of a bispecific IgG, we evaluated three additional disulfide bond positions, all in the structurally conserved framework region of the Fv molecule with short Cα-Cα distances and side-chain orientation. The positions were L98-H45, L43-H105 and L43-H106, previously tested in the context of dsFvs. Symmetrically engineered “disulfide-stabilized IgGs” (see Figure 2B) based of the anti CD30 antibody T427 were constructed using these cysteine mutations and were compared to WT and L100-H44 IgGs. As shown in Figure S2, none of the newly evaluated cysteine pairs equaled the performance of WT or the L100-H44 antibodies, primarily because they failed in the refolding/assembly process (Figure S2A) and as evident by their compromised binding avidity (Figure S2B). These results confirmed the superiority of the L100-H44 disulfide bond position in the context of a refolded bispecific IgG, similar to what was previously reported for dsFvs [23,30].

### 3.6. Evaluation of CH1-CL Positions for Disulfide-Stabilization of the Fab Arm Interface of bsAbs

**Figure 8:**
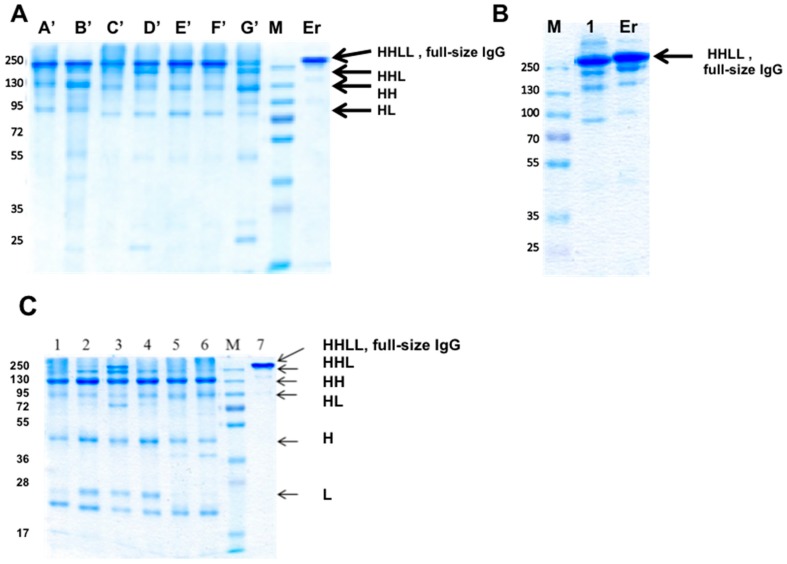
Evaluation of bsAb assembly by SDS/PAGE. (**A**) The purified “disulfide-stabilized IgGs” engineered at the CH1-CL interface; molecules engineered on the T427 Fab arm (Kappa light chain): A-G represent the seven bsAb mutants as listed in Table 1. (A’) Heavy chain T427 F174C, C222A; Light chain T427 S162C, C214Δ; (B’) Heavy chain T427 F174C, C222A; Light chain T427 T164C, C214Δ; (C’) Heavy chain T427 L124C, C222A; Light chain T427 F118C, C214Δ; (D’) Heavy chain T427 F122C, C222A; Light chain T427 S121C, C214Δ; (E’) Heavy chain T427 F122C, C222A; Light chain T427 E123C, C214Δ; (F’) Heavy chain T427 F122C, C222A; Light chain T427 Q124C, C214Δ; (G’) Heavy chain T427 S130C, C222A; Light chain T427 F118C, C214Δ; (M) protein size marker (MW in kDa); (**B**) A bsAb engineered on the anti SA Fab arm (Lambda light chain): 1 αSA IgG carrying mutations Heavy chain Q175C, C222A and Light chain E160C, C214A. Er-Erbitux^®^ shown as a reference to full-size IgG. The arrows indicate the different chains composition in the sample, where L = Light chain and H = Heavy chain; (**C**) Analysis of the “illegitimate pairing” family. All mutants consist of Heavy chain T427 WT + a mutant of Light chain as follows: C214Δ + 1) F118C (light chain of mutant C from Table 1); (2) S121C (light chain of mutant D from Table 1); (3) E123C (light chain of mutant E from Table 1); (4) Q124C (light chain of mutant F from Table 1); (5) S162C (light chain of mutant A from Table 1); (6) T164C (light chain of mutant B from Table 1); (7) Erbitux^®^ (used as a full-size IgG protein marker). The arrows mark the different chains composition in the samples, where L = Light chain and H = Heavy chain. The purified antibodies were separated on a 10% SDS-PAGE under non-reducing conditions and visualized using GelCode Blue™ staining.

To evaluate the potential of interfacial positions in the CH1-CL interface, we prepared 7 “disulfide-stabilized IgGs” (mono-specific disulfide stabilized antibodies, molecule B in Figure 2); these mAbs were symmetrically engineered on both Fab arms with artificial disulfide bonds, replacing the naturally-occurring disulfide bond (mutations HC222A and LC214Δ). The “disulfide-stabilized IgGs” were prepared to evaluate chain pairing of the Fab arm and were all based on an unmodified human IgG1 Fc. The antibodies were produced by refolding and purified by MabSelect affinity chromatography followed by SEC on a Superdex200 column. Fractions of the purified bsAbs were evaluated by SDS/PAGE, as shown in Figure 8A. In Figure 8B we evaluated a “disulfide-stabilized IgG” antibody based on anti SA.

As shown, fully assembled IgGs formed for all the molecules, suggesting that all the seven position pairs allow the assembly of a full-size IgG in T427 and that the position pair tested with αSA allowed chain pairing and assembly of a full-size IgG as well.

To investigate whether “illegitimate pairing” (meaning, assembly of a full-size IgG by combining WT H chains with engineered L chains) can occur, an additional set of antibodies were prepared, where a WT T427 H chain was refolded with mutated L chains. As shown in Figure 8C, IgG assembly was mostly inhibited, but not entirely. Since these H–L chain pairs cannot form covalent bonds, they should migrate as H chain pairs and free light chains (T427 has a very stable H–L interface, which allows H–L pairing even without an inter-chain disulfide bonds. However, upon separation by SDS/PAGE, the L chains are separated from the H chains. In the molecules shown in Figure 8C, fully assembled and ¾ IgGs (HHL) can be observed, suggesting incomplete prevention of “wrong” chain pairing.

### 3.7. Using “Destructive Mutations” to Prevent “Illegitimate” H–L Chain Pairing

From the experiments described above, and evaluation of bsAbs that were constructed according to our design principles but expressed in transfected mammalian cells (see below) we learned that the position pairs we chose for placing cysteine residues, whether the Heavy chain C44 with Light chain C100 or the CH1-CL positions shown in Figure 8A, all allowed the assemble of full size IgGs. However, only the H chain C44 combined with L chain C100 pair prevented illegitimate pairing, while such pairing was not fully inhibited for the CH1-CL molecules. We hypothesized that we may be able to prevent illegitimate pairing by adding mutations that will prevent chain pairing to some extent, allowing the assembly of full-size IgG only when the artificial disulfide bond holds the H and L chain covalently attached. We named these “destructive mutations”. To test this hypothesis, we prepared a set of antibodies that carried an artificial disulfide bind in the Fab arm, but where the natural cysteines that form the inter-chain disulfide bond in WT antibodies were replaced by arginine or tyrosine instead of alanine which is used in our ordinary design.

**Figure 9:**
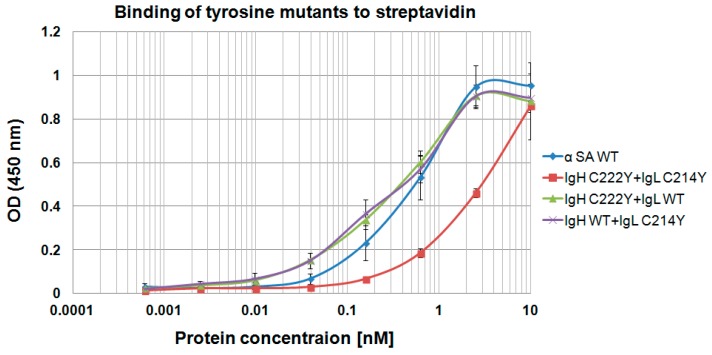
Evaluation of “disulfide-stabilized IgG”, anti SA “tyrosine mutants” binding to streptavidin by ELISA. Binding capability was evaluated by ELISA with SA as the coated antigen using X4 dilutions starting at 10 nM of each examined antibody; 3% skim milk was used for blocking; binding was detected using HRP-conjugated goat anti-human antibody (diluted 1:5000 in PBST). Error bars represent the standard deviation of the data.

Of note, our model antibodies, T427 and αSA are ones where the H–L chain association is a tight one, allowing the assembly of a full-size IgG even when the H–L inter-chain disulfide bonds are eliminated, so there is no covalent bond holding the chains together. When we replaced the cysteines that form the inter-chain disulfide bonds with arginines or with tyrosines, H–L chain assembly was still obtained with arginines (Figure S3), but not with tyrosines. We cannot show SDS/PAGE analysis of the tyrosine mutants, as no antibody could be obtained upon refolding of the corresponding Heavy chain αSA C222Y+ Light chain αSA C214Y mutant (it aggregated during refolding, suggesting severe destabilization). Since MabSelect was used to purify the antibodies shown in Figure S3, the fact that light chains can be observed in the gel suggests that all the mutants assembled as full-size IgGs with no inter-chain H–L covalent bonds. This was further supported by evaluating the binding of these IgGs to streptavidin by ELISA. As shown in Figure S1, anti SA is an antibody where both the H and the L chain contribute to binding, and the absence of either one impairs binding severely. The similar EC_50_ values shown in Table 2 further suggest that full-size, binding competent anti SA IgGs were assembles for the tested mutants. This was the case when alanine or arginine replaced cysteine 222 of the H chain or C214 of the light chain, or both, suggesting that such replacements did not provide sufficient repulsion of the H and L chains to prevent IgG assembly. As a matter of fact, only when tyrosines were used to replace both cysteine 222 of the H chain or C214 of the light chain. When tyrosine was used on one of the chains, IgG assembly was still permitted. This is evident from the ELISA shown in Figure 9.

**Figure 10:**
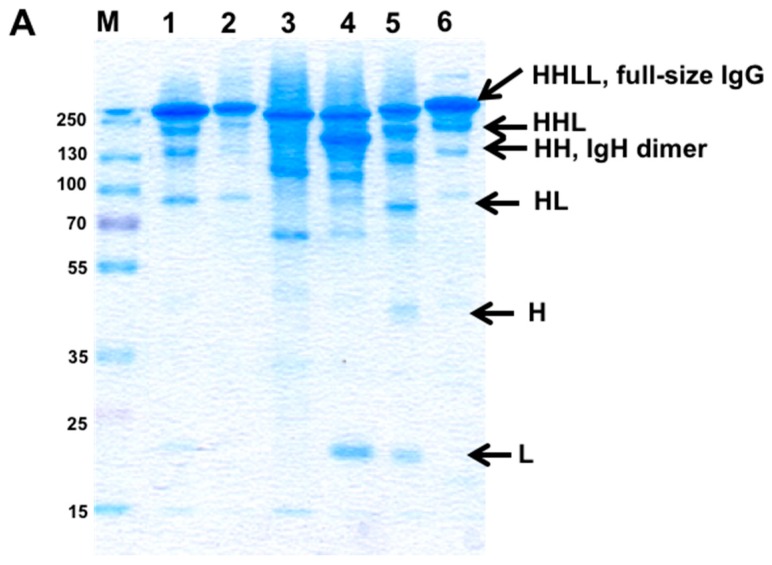
Evaluation of “disulfide-stabilized IgGs” and bsAbs containing tyrosine mutations by SDS/PAGE. 1. Heavy chain αSA Q175C C222Y + Light chain αSA E160C C214Y; 2. T427 WT (Heavy chain T427 WT + Light chain T427 WT); 3. Heavy chain T427 G44C C222Y + Light chain T427 G100C C214Y; 4. BsAb: αSA-Heavy chain Q175C C222Y hole + Light chain E160C C214Y and T427-Heavy chain G44C C222Y knob + Light chain G100C C214Y; 5. BsAb: T427-Light chain G100C C214del and Heavy chain G44C C222A knob + αSA Light chain and Heavy chain-hole chains; 6. Erbitux (used as a full-size IgG protein marker). 5 µg of each purified antibody were separated on a 10% SDS-PAGE under non-reducing conditions and visualized using GelCode Blue™ Staining.

The double mutant C222Y + C214Y (also abbreviated YY) shown in Figure 9 is not accurately evaluated, since as described above, we could not purify it as a monomeric species after refolding as it mostly aggregated, so we used unpurified crude refolding solution to evaluate it. In conclusion, in this family of tyrosine mutations, interference of H–L chain association was obtained only when Y in the heavy chain was facing Y in the light chain, which resulted in mainly aggregative fraction and with reduced binding ability to streptavidin. The other combinations of Y in the heavy chain facing C in the light chain and vice versa did not prevent the assembly of the antibody to a full-size IgGs nor did it impair their binding ability. Therefore, we realized that in contrast to the H chain C44 with L chain C100 combination in which one side of the bispecific antibody is WT at the Fab-arm interface while the second side in engineered, the tyrosine solution for preventing illegitimate chain assembly will have to involve engineering of both Fab arms of the bsAb with tyrosines. Indeed, when artificial disulfide bonds were used in context of the double mutant C222Y + C214Y, fully assembles and binding competent bsAbs could be obtained, as shown in Figure 10.

To evaluate how these mutations affected the thermal stability of the resulting antibodies, we carried out Nano-DSF measurements on a selected set of mutants that were produced in transfected mammalian cells (see below). Tm2 values that were obtained during these experiments represent the melting temperature of the Fab arm. These nanoDSF measurements were carried out on T427-αSA bsAbs where the engineered Fab arm was the T427 side. The results are shown in Table 3. All the bsAbs shown in Table 3 have a KIH Fc and the naturally-occurring inter-chain H–L disulfide bond eliminated, so only the positions of the cysteines forming the artificial disulfide bond are shown in the table. Being bsAbs, we expected the Tms to be an average of those of the parental mAbs, which appears to be the case. What is most interesting is that the introduction of the “destructive” YY mutations had a small effect on the Tm2 values (reduction of about 2 °C). Suggesting that for a disulfide-bonded Fab arm, the stabilizing effect of the artificial inter-chain disulfide bond largely overcomes the destabilizing effect of the YY mutations.

Finally, the experiments described so far that were carried out using IgG refolding were carried out to establish the suitability of various antibody sequences to promote (or prevent) H–L chain assembly. As is frequently the case with refolded antibodies, they were not produced with high yield, were not of particularly high purity nor did they achieve high % of full IgG assembly. These parameters were addressed with bsAbs constructed according to our design principles, but expressed in transfected mammalian cells, as described below.

### 3.8. Production of bsAbs in Transfected Mammalian Cells

The results presented so far were of mono and bispecific IgGs obtained by bacterial expression and refolding. To test if our design principles for bispecific IgGs apply also for antibodies that are expressed in mammalian cells, several experiments were carried out. In these experiments three designs were evaluated; the positions of the artificial disulfide bonds that stabilized the engineered Fab arm interface were: *H* (Heavy chain C44/Light chain100) or *B* (Heavy chain C174/Light chain C164) or *E* (Heavy chain C111/Light chain C123). bsAbs were produced using the Expi293™ transient transfection system. Antibodies were purified from conditioned media collected 4–6 days post transfection. Purification was either by a single affinity chromatography step (using MabSelect or KappaSelect affinity columns) or when chain assembly of the secreted IgG was not optimal, by sequential KappaSelect followed by LambdaSelect chromatography. The production yield of mAbs produced using the Expi^293^ system ranges between 15 to 300 mg/L conditioned medium by a single chromatographic step in our hands. There is a very large difference in the production level of different mAbs of different sequences of the variable domains. Our experience with bsAbs is that the production yield is roughly similar to that of the parental mAb which is expressed less efficiently that the second parental mAb. When two sequential affinity purification steps are required, the final yield is about 20% of a that obtained by a single purification step. Production yields of the antibodies that were described in Table 3 are described in Table 4. The bsAbs were produced by transient transfection of Expi293TM cells and purified from 30 mL of conditioned media by sequential KappaSelect followed by LambdaSelect affinity chromatography. The mAbs were purified by a single MabSelect affinity chromatography step.

**Figure 11:**
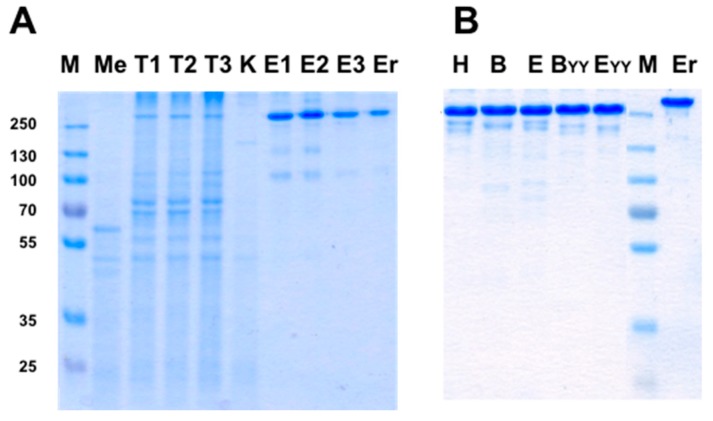
Analysis of the bsAbs produced in Expi293™ mammalian cells by SDS/PAGE. (**A**) 3 independent transfections used to produce a bsAb with an WT T427 Fab arm and an engineered anti CD24 Fab arm (the anti CD24 antibody is a humanized IgG1-Kappa). Me, conditioned medium of untransfected cells; T1, T2, T3 are day 6 conditioned media from 3 transfections. K, KappaSelect unbound fraction; E1, E2, E3 are the bsAbs eluted from the KappaSelect column. Er, Erbitux (used as a full-size IgG protein marker); (**B**) bsAbs identical to those described in Table 3 above. 2.5 µg of each of the purified antibodies were separated on a 10% SDS-PAGE under non-reducing conditions and visualized using GelCode Blue™ staining.

The purity of several bsAbs, including the ones described in Table 4 is shown in Figure 11. As shown, it is possible to obtain highly pure bsAbs with a single step affinity purification (Figure 11A) or by sequential two affinity chromatography steps (Figure 11B). We estimate the purity level of the antibodies shown in Figure 11A at >90% and in Figure 11B at >95%.

### 3.9. Evaluation of Binding Affinity by SPR

The kinetic parameters and the affinities for CD30 and SA of the bsAbs *H*, *B*, *E*, *BYY* and *EYY* (described in Table 3) and the parental mAbs T427 and αSA were determined by surface plasmon resonance (SPR) and are presented in Table 5. The equilibrium dissociation constants, K_D_ values, were calculated as the ratio of the antibody dissociation rate (k_d_), to the antibody association rate (k_a_): k_d_/k_a_. All the calculations were performed using a kinetics model based on 1:1 binding. This explains the differences between the values of the bsAbs compared with their parental antibodies, due to their different avidity. Therefore, the summarized constants are empirical and not mechanistic. All the tested bsAbs showed comparable binding ability to each of the antigens and they all bind in the nanomolar range, thus the mutations in the Fab arm did not significantly affect their binding ability (except for the *EYY* mutant whose affinity to SA was somewhat reduced). As for the binding to CD30, all the tested antibodies demonstrated a comparable binding ability.

### 3.10. Large-Scale Production in CHO Cells

To scale up production and further study expression of bispecific IgGs in mammalian cells, we contracted a certified CRO to carry out large scale stable expression. The model antibody was an anti vascular endothelial growth factor (VEGF)—anti angiogenin 2 (Ang2) bsAb identical in sequence to the one that was tested by Roche during the evaluation of the “Crossmab” technology [9]. When expressed according to our “BIClonals” design (artificial disulfide bond at positions Heavy chain C44/Light chain 100), the anti VEGF side (sequence based on the commercial mAb bevacizumab, human IgG1-κ light chain) carried the “hole” mutations in C_H_3 and was engineered in the Fab arm interface while the anti Ang2 antibody (based on the sequence of Roche antibody LC06 [9], human IgG1-λ light chain) carried the “knob” mutations in C_H_3 and was unmodified at the Fab arm interface. The antibody was expressed by retroviral transduction of CHO cells which were kept as a cell pool. For comparison, the same bsAb was also produced by refolding. The refolded bsAbs (produced in-house) was named BIC101 and the mammalian cells produced bsAb was named BIC201.

**Figure 12:**
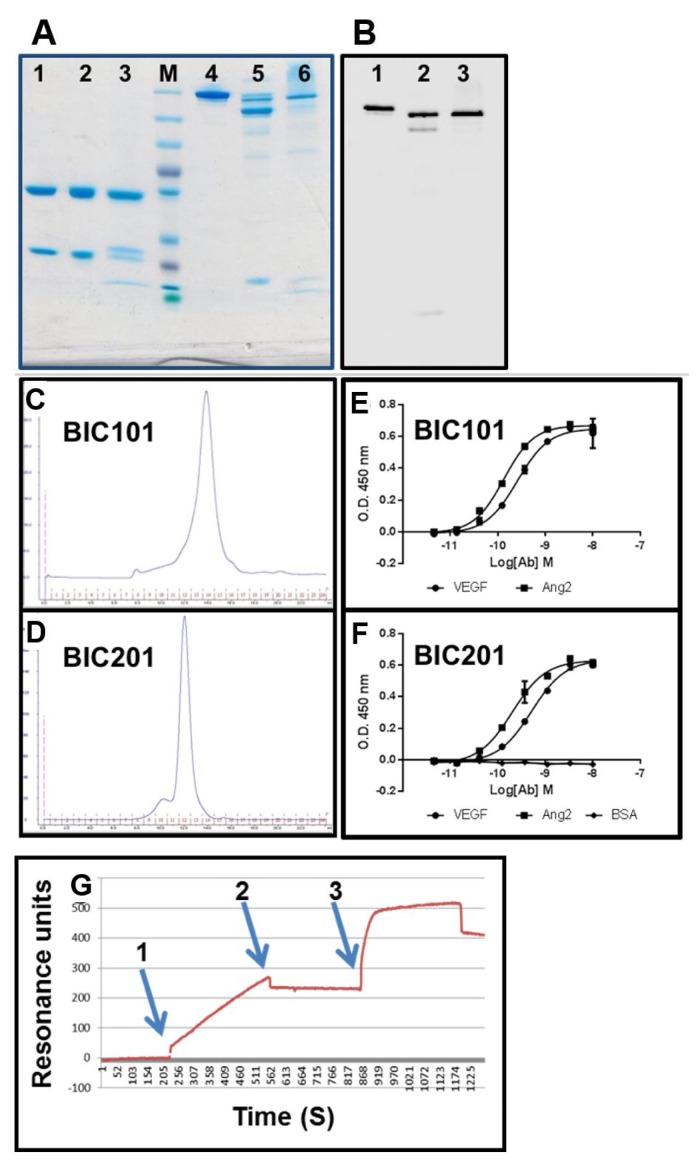
Production and evaluation of an anti VEGF—anti Ang2 bispecific IgG produced by refolding and in CHO cells. (**A**) Samples of purified BIC101 (made by refolding) and BIC201 (made in CHO cells) were analyzed by SDS/PAGE. Lanes 1 and 4, the commercial mAb bevacizumab (Avastin), lanes 2 and 5, BIC201, lanes 3 and 6, BIC101. 3–5 μg were loaded per lane of a 10% acrylamide gel, samples in lanes 1–3 were separated under reducing conditions while those in lane 4–6 were not reduced; (**B**) Immunoblot of bevacizumab (lane 1), BIC201 (lane 2) and BIC101 (lane 3). Antibodies were separated as in lanes 1–3 of (**A**), electro-transferred to nitrocellulose and detected using an anti-human kappa light chain antibody; (**C**) analysis of BIC101 and (**D**) analysis of BIC201 by analytical size-exclusion chromatography on a Superdex200 column. 100 µg of each bsAb were injected into the column that was developed using PBS as the mobile phase at 1 mL/min; (**E**) evaluation of antigen binding by BIC101 and (**F**) by BIC201 by ELISA. Shown are composite graphs combining separate binding assays carried out on each antigen. Binding was detected using HRP-conjugated goat anti-human antibody (1:5000). Error bars represent the standard deviation of the data; (**G**) evaluation of BIC201 bispecificity by SPR (carried out essentially as described in method S6). VEGF was immobilized onto a CM5 sensor chip and the antibody was flowed over the chip for 300 s. At this point, buffer was flowed over the chip for another 300 s, followed by 300 s of Ang2.

Analysis of BIC101 and BIC201 by SDS/PAGE, immunoblotting and analytical SEC can be seen in Figure 12. As shown, the bsAbs were efficiently produced as fully assembled IgGs, with BIC201 showing more partially-assembled species than BIC101. The SEC profiles (Figure 12C,D) suggest that they eluted mostly as monomers from the Superdex 200 column. BIC201 has a higher MW according to the SEC analysis, probably due to the fact that it is glycosylated while the *E. coli* produced BIC101 is not. Analysis of VEGF and Ang2 binding by ELISA is shown in Figure 12E,F. As shown, BIC101 and BIC201 bound both antigens with very similar sub-nanomolar affinities (VEGF EC_50_ of 0.26 nM for BIC101 and 0.52 nM for BIC201 Ang2 EC_50_ of 0.14 nM for BIC101 and 0.2 nM for BIC201), which are also similar to those reported for the Crossmab version of this bsAb [9]. SPR was used to confirm the affinity estimation of the ELISA (not shown) and for establishing the bispecific structure of BIC201. As shown in Figure 12G, a binding signal was obtained when BIC101 was flowed over a sensor chip that has been coated with VEGF (Figure 12G arrow 1). When the flow of BIC101 was terminated (Figure 12G arrow 2) and Ang2 was then flowed over the sensor chip (Figure 12G arrow 3), an increase in the binding signal was observed, establishing that, indeed, BIC201 is a true bsAb.

### 3.11. Study of the Blood Pharmacokinetics of BIC101 and BIC201 bsAbs Made in CHO Cells in Mice

**Figure 13:**
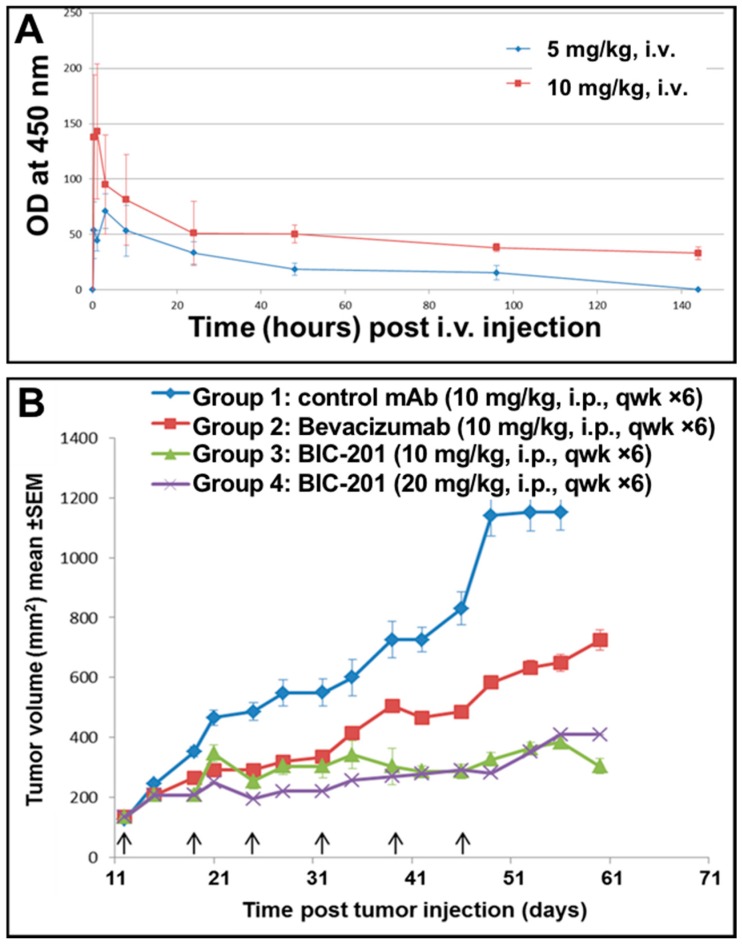
Mouse studies of BIC101 and BIC201. (**A**) Mouse blood pharmacokinetics of BIC101 and BIC201. Mice were injected with a single dose of 5 mg/kg of BIC101 (blue diamonds) or 10 mg/kg of BIC201 (red squares). The concentration of the bsAbs in the mice sera was evaluated by ELISA using human Ang2 as an antigen; (**B**) Bispecific anti Ang-2–anti VEGF BIC201 inhibits the growth of Colo205 tumor xenografts compared to the monotherapy of bevacizumab. The anti CD20 therapeutic mAb rituximab was used as isotype control (group 1). Antibodies were administered i.p. (*n* = 10 mice per group). Treatment started at day of randomization (study day 12) with 10 mg/kg or 20 mg/kg once weekly for a total of 6 injections (indicated by vertical arrows).

The blood pharmacokinetics of BIC101 and BIC201 were tested following IV injection to CD-1 mice. As shown in Figure 13A, both molecules behaved very similarly in their PK parameters, with a calculated t_1/2_ of about 3 days.

A colo205 xenograft study was carried out to evaluate the potency of BIC201. As shown in Figure 13B, significant tumor growth inhibition could be observed for mice treated with the anti VEGF bevacizumab at 10 mg/mg (Tumor growth inhibition (TGI) of 41% on day 24 of the study). More significant tumor growth inhibition could be observed in mice that were treated with BIC201 at 10 (TGI of 64% on day 24 of the study) or 20 mg/kg (TGI of 78% on day 24 of the study) compared with the control group (* *p* < 0.05 compared with control group, by student’s *t* test). The extent of tumor growth inhibition shown in Figure 13B is similar to that reported for Crossmab antibodies constructed from the same anti VEGF and anti Ang2 arms [9]. These results provide additional support to the success of our design principles for bispecific IgGs to yield fully functional bsAbs.

## 4. Discussion

The bsAb R&D arena is intense and the growing interest in the therapeutic potential of bsAbs over the past few years resulted in a plethora of bsAb formats [2,31,32]. In many cases, due to issues such as of poor manufacturability, PK/PD properties or immunogenicity, such formats may prove ineffective for therapeutic applications, resulting in a high attrition rate. We believe that the IgG format that has been selected during evolution has many advantages for the therapeutic application of mAbs in general and of bsAbs in particular. These advantages include optimal pharmacokinetic properties, retained effector functions such as ADCC and CDC and excellent stability and solubility. Many of the bsAb formats being evaluated are indeed based on IgG scaffolds, in many cases to which additional binding modules such as peptides or small antibody fragments are fused [2,8]. There are only a few bsAbs that are truly faithful to a native IgG architecture and even fewer that can be produced by a single antibody expressing cell [8,11]. 

The main challenges for making bispecific IgGs in a single producing cell are referred to as “overcoming the chain association issue” [8], or making sure that when two different heavy chains and two different light chains are co-expressed within a cell, only the correct ensemble of two different heavy chain, each bound to its cognate light chain will be allowed and all other possible chain combinations will be prevented [8,11]. We believe that the “BIClonals” design principles for bispecific IgGs we present here meet these challenges.

We do not claim any credit for choosing the KIH solution for H–H heterodimerization. This elegant solution was presented by Genentech 22 years ago with the mapping of the KIH mutations in the C_H_3 domains of human IgG1 [12] and further modified to include a stabilizing artificial disulfide bond, also between the C_H_3 domain, resulting in even better correct chain association [10]. The KIH solution is still the most commonly used solution for heavy chain heterodimerization applied for bispecific IgG and IgG-like bsAbs. KIH was initially demonstrated for linking different heavy chains in transfected mammalian cells [10], and several years later was also demonstrated for bacterially expressed antibodies, where the two heavy chains are individually secreted in two separate bacterial cultures, followed by in vitro assembly of the antibody [33] or by co-culturing to allow heavy chain heterodimerization to occur [34]. Of note, our report is the first example of refolding a bispecific IgG based on KIH heavy chain heterodimerization. While we make no claims regarding the scale-up potential of IgG refolding and its potential for making therapeutic mAbs at an industrial scale, we found it to be a rapid and convenient tool for evaluating antibody assembly and functionality. Indeed, using refolding we could show: (1) correct formation of KIH–stabilized heavy chains and prevention of formation of “wrong” heavy chain pairs (see Figure 3 and Figure 4); (2) correct formation of H–L chain pairs, involving both κ and λ light chains (see Figure 5 and Figure 6) and the formation of a fully-assembled bispecific human IgG1 that could recruit an effector molecule (a toxin) by one arm to target model cancer cells that display the target of the other arm (see Figure 7).

During the past 10 years or so, additional solutions were presented for heterodimerization of human heavy chains. A few examples for solutions that do not involve using non-antibody associating domains to facilitate heterodimerization (such as “Dock and lock” [16] or coiled-coils [35]) are “SEEDbodies” involving strand-exchange between human IgG and IgA C_H_3 domains [36], “electrostatic steering” involving placing residues of opposite charge on the two heavy chains [37], IgG1-IgG2 heterodimerization of in vitro assembled bsAbs [38] and IgG4 hinge-based “Fab arm exchange” [39]. Our “BIClonals” solution for correct H–L pairing, being orthogonal to H–H chain heterodimerization can most probably be applied in combination with any existing solution for heavy chain heterodimerization.

Concerning the correct H–L pairing issue, the picture narrows as many solutions for heavy chain heterodimerization within a single antibody producing cell were demonstrated using a single light chain that associated with both heavy chains. This solution is not ideal, as it does not provide an opportunity to derive bsAbs from any existing mAb in a straightforward manner. Examples for solutions that truly fulfill all the criteria for an IgG-like bsAb, with two different heavy chains and two different light chains, all assembling properly within a single producing cell are Roche’s “Crossmab” technology, where correct association of the light chains and their cognate heavy chains is achieved by exchange of heavy-chain and light-chain domains within the Fab of one half of the bispecific antibody [9,40], “Duetmab” technology published by Medimmune in 2015 [14,41], which like our solution, involves replacing the native H–L inter-chain disulfide bond by an artificial CH1-CL inter-chain disulfide bond and Eli-Lilly’s “KIH like” approach involving the engineering of the Fab arm interface to design an orthogonal IgG heavy chain–light chain interface using molecular modeling with feedback from X-ray crystallography [11]. We suggest that our “BIClonals” solution is effective in allowing the assembly of bsAbs that are truly IgG-like with the minimal number of point mutations (four) introduced into the Fab arm.

A critical requirement for correct H–L pairing is that, not only will correct H–L pairs be allowed but also that the formation of “wrong” H–L pairs be prevented. To evaluate to what extent our solution fulfills this requirement we carried out the experiment described in Figure 6. In these experiments we evaluated the formation of correct and wrong H–L chain pairs using symmetrically-engineered mAbs. According to the SDS/PAGE analysis, both the T427 mAb (IgG1-κ) and the anti-SA mAb (IgG1-λ) could be obtained as fully assembled IgGs only when either two WT H and L chains or when two engineered H and L chains were combined. In contrast, when a WT heavy chain was combined with an engineered light chain or vice-versa, the assembly of full-size IgG was impaired. The antibodies presented in Figure 6 were purified by protein-A chromatography only after refolding. When IgGs are prepared by refolding, the total yield (how much purified monomeric antibody is obtained) and preparative SEC (separating monomers from aggregates) provide important tools for evaluating correctly refolded IgGs. In the experiment shown in Figure 6, we studied the formation of H–L pairs where the engineered anti-SA heavy chain was combined with an engineered light chain or with WT light chains of anti-SA (λ) or of T427 (κ). Refolding of the four antibodies was initiated at 500 mL scale (using 25 mg of heavy chain inclusion bodies and 25 mg of light chain inclusion bodies). Following refolding, the antibodies were purified by MabSelect affinity chromatography followed by preparative gel filtration. The recovery yields were about 3–5 mg of monomeric WT or fully engineered IgGs and less than 200 micrograms of “wrong pair” IgGs, suggesting very poor association of wrong H–L pairs. When the complete eluate of the MabSelect column was loaded on the SEC column, the elution profiles shown in Figure 6A suggest that the WT and fully engineered IgGs were produced with similar efficiency. As shown in Figure 6B, they also bound SA with similar avidity. In contrast, the “wrong pairs” molecules were formed with much lower efficiency and their SA binding avidity was severely impaired. The mere addition of the V_H_ Cys44 mutation (of the V_H_ C44 V_L_ WT (λ) shown in Figure 6) is sufficient to impair the antibody assembly with a WT light chain and its functionality. These results, and the fact that such chain pairs failed to assemble also upon expression in mammalian cells (Figure 6C) suggest that not only do the engineered cysteine residues located at the V_H_–V_L_ interface facilitate the formation of the artificial disulfide bond, they actually clash with the corresponding interfacial residue of the WT light chain (coming from the non-engineered side of the bsAb) thus fulfilling the two critical criteria for correct H–L pair formation. We believe this is due to the nature of interfacial residues located at these positions (H44 and L100) of variable domains of human antibodies [42]. V_H_ position 44 is mostly occupied by an amino acid with a small side chain (glycine is most common, appearing in 44/50 human germline genes with alanine appearing 4 time and arginine appearing twice) while V_L_ position 100 (located in Jκ and Jλ) is occupied by glutamine (4 of 5 Jκ) proline (1 of 5 Jκ) threonine (1 of 7 Jλ) glycine (4 of 7 Jλ), serine (1 of 7 Jλ) and glutamic acid (1 of 7 Jλ) [16]. We hypothesize that when a cysteine is inserted at these positions, a very important interfacial interaction that stabilizes the V_H_–V_L_ interface is disrupted or a clash is formed. Interestingly, when we evaluated positions for disulfide stabilization of the Fab arm using artificial disulfide bonds between C_H_1 and C_L_, we found that these engineered cysteines do not fully inhibit “wrong” H–L formation, and additional mutations were required to fulfill this requirement (the YY mutants). Our results are in agreement with the observation of Lewis et al. in the study of structure-based design of an orthogonal Fab interface [11] who stated “… we have found that V_H_–V_L_ interactions can dominate the interaction specificities between heavy chains and light chains”.

Our choice of positions for introduction of the cysteine mutations that formed the artificial disulfide bond that replaced the native inter-chain disulfide bond was inspired by studies of disulfide-stabilized Fv fragments (dsFvs) that were initiated in 1992 [21,22,23,29,43]. During those studies, a number of position combinations were evaluated computationally [42], some were also evaluated experimentally for their potential to form disulfide bonds. These positions were located in the V_H_–V_L_ interface form between the FR2 region of V_H_ to the J domain of V_L_, or, because of the “pseudo symmetry” of Fvs, between residues in the J_H_ domain and the FR2 region of the V_L_ domain. The selection criteria for positions were being located in structurally conserved framework regions, being remote from the CDR loops that make up the binding site of the antibody and located at suitable Cα-Cα distances that correspond to typical disulfide bonds [30,42]. In the context of dsFvs, a few combinations were successful in generating functional antibodies. The V_H_ C44–V_L_ C100 combination was the most successful and most commonly applied solution [21,22,23,29,42,43] which was the reason we chose it for disulfide stabilization of the Fab arm interface of “BIClonals”. To check if other position pairs that could stabilize dsFvs could be also used for making bispecific IgGs, we chose three additional pairs and compared IgGs that contains those with IgGs built according to the L100-H44 design. As shown in Figure S2, the IgGs that were constructed based on positions L98-H45, L43-H105 and L43-H106 failed to assemble as full-size IgGs (Figure S2A) and bound CD30 quite poorly compared to the L100-H44 IgG (Figure S2B). These results suggest that while dsFvs may tolerate more than a single solution for disulfide stabilization, an IgG is less tolerant. Still, we cannot rule out that for other antibodies, or for bsAbs produced in mammalian cells, additional positions for disulfide stabilization could be functional. The most comprehensive list of potential position pairs can be found in [44].

With regard to artificial disulfide bonds located in the CH1-CL interface, we selected several position pairs to study based on 3D models of IgGs. Interestingly, all the position pairs we tested allowed the formation of full-size IgGs (see Figure 8). However, different from our bsAbs where the artificial disulfide bond links the V_H_ and V_L_ domains, the inhibition of wrong chain pairing was not complete with some of these antibodies. To more effectively inhibit wrong chain pairing, we had to introduce the “destructive” YY mutations and to engineer both Fab arms of the bsAbs. An additional interesting observation was that the introduction of the “destructive” YY mutations resulted in a modest decrease in the melting temperature of the Fab (see Table 3). Apparently, the covalent linking of the H and L chains by the artificial inter-chain disulfide bonds largely overcame the repulsion between that chains resulting from the opposing tyrosines. Thus, artificial disulfide bonds linking the CH1-CL domain provides an effective solution, but we have a personal preference for the Heavy chain C44/Light chain C100 solution because it involves the engineering of only one Fab arm, thus requiring only 4 mutations in the Fab arm.

As for the legitimacy of using artificial disulfide bonds to stabilize IgGs, perhaps the first example was the “second generation KIH” [12] that differed from the “first generation” [10] by the addition of an artificial disulfide bonds between CH3 positions 349 of the “hole” heavy chain and 354 of the “knob” heavy chain. This provided extra robustness to the KIH bsAbs, improving their assembly efficiency. Artificial disulfide bonds have been used to stabilize immunoglobulin domains in “Fcabs” (Fc domains with engineered loops creating antigen binding sites in constant domains) [45,46]. As for artificial disulfide bonds used to prepare bispecific IgGs, as mentioned above, Medimmune’s “DuetMab” technology is very similar to our set of bsAbs with an artificial disulfide replacing the native one in the CH1-CL interface. In their publications, Mazor et al. showed convincingly that wrong H–L chain pairing does not occur with their molecules and that such bsAbs maintain stability [14,41]. There is a difference in that, while we replaced the cysteines of the native disulfide bonds by Alanine, Mazor et al. replaced them with glutamines. This could be a reason for better chain assembly, or the very important fact the H–L pairing is driven largely by interfacial V_H_–V_L_ interactions [11], so different antibodies may assemble to differing extent using very similar design principles. An additional relevant comment is that even in cases where complete assembly of full-size bispecific IgGs does not occur during expression within the single cell that expressed 4 recombinant proteins, as long as the correctly assembled IgG is the dominant species, it can be separated from contaminating partially-assembled species by chromatography. This is what we do, by using sequential KappaSelect–LambdaSelect chromatography (suitable for antibodies where one arm has a kappa and the other a lambda light chain). Still, the design of the pairing strategy should be sufficiently robust to satisfy developability requirements.

Therapeutic IgGs are usually produced in cultured mammalian cells. To evaluate if the “BIClonals” design principles apply for bispecific IgGs expressed in mammalian cells, we expressed them in small scale in transiently-transfected Expi293™ cells and in large scale in retroviral-vector transfected CHO cells. As shown in Figure 6C and Figure 11, our design principles were effective in allowing the assembly of full-size IgGs while combinations of “wrong” pairs resulted only in partially assembled species.

An approach that may be suitable for studying H–L chain assembly is mass-spectrometry, where the intact mass of a bsAb can identify the H and L chains in the molecules. Such an approach was used to study chain pairing variants of bispecific IgGs Expressed in a single host cell [47,48]. Mazor et al. used tandem mass spectrometry (MS-MS) to study chain pairing in “Duetmabs” [14]. We attempted a different approach, usually used to study protein complexes, chemical crosslinking followed by tandem mass spectrometry (CL-MS) [49,50,51]. However, the crosslinked species we could identify were not informative for identifying which heavy chain is crosslinked to which light chain. We are not aware of CL-MS being used to study chain pairing of antibodies and while seemingly a very powerful and high-resolution approach, further optimization is required to make it useful for studying antibodies. 

The anti VEGF—anti Ang2 bispecific IgG was chosen as a proof-of-principle for large scale production since this sequence was evaluated as a bsAb according to the “Crossmab” technology [9]. In addition to its production in CHO cells (BIC201), the same bsAb was also produced by refolding according to the “Inclonals” protocol [17] as modified herein for the production of bispecific IgGs (BIC101). The production of BIC201 in the CHO expression system was comparable in yields and quality to conventional IgG antibodies and it could be purified using standard IgG purification procedures. As shown in Figure 12, both BIC101 and 201 could be produced as monomeric proteins, showing binding affinity values similar to those reported for the corresponding Crossmab as reported in the literature [9]. Of note, the product of transduced cell pool contained a significant amount of partially-assembled antibodies (mostly 2 heavy chains with a single light chain). We suggest that a fully assembled IgG could be obtained by increasing the light chain gene dose in such transductants (with this retroviral vector transduction it is possible to increase the gene dosage incrementally), or by selecting single cell clones by limiting dilution to establish a master cell bank of cells that produce a fully assembled antibody. Another possibility is to use sequential chromatography using Kappa-select followed by Lambda-Select affinity chromatography columns (General Electric (GE) healthcare, Boston, MA, USA) (or in a reverse order of columns) to obtain only fully assembled IgGs with two different light chains [52].

When the blood pharmacokinetics of BIC101 and BIC201 was evaluated in mice, the two molecules behaved quite similarly with a t_1/2_ of about 3 days, which is typical of human IgG1 antibodies in mice [35,53]. Of note, although BIC101, being produced in bacteria is aglycosylated while BIC201 is glycosylated, binding to the FcRn receptor (which is the dominant factor for determining serum half-life) is not affected by glycosylation [54]. To evaluate potency, a Colo205 xenograft study was carried out. As shown in Figure 13, BIC201 given six times, once weekly i.p. at doses of 10 or 20 mg/kg inhibited tumor growth significantly more than bevacizumab (which corresponds to the anti VEGF arm of BIC201) given at 10 mg/kg. We did not explore in depth the efficacy of the anti Ang2 arm alone or the combination of the mAbs that have been investigated in [9] since the emphasis in our mouse study was to compare our first bsAb to be produced in large scale in CHO cells to an already established bsAb and to demonstrate technical feasibility of this approach as a proof-of-principle for our design principles. Indeed, our results are similar to what was reported for the Crossmab version of that antibody [9] and further suggest that the “BIClonals” design principles for bispecific IgGs offer a genuine solution for effective construction of bsAbs.

To conclude, we present a solution for making bsAbs in a single antibody expressing cell which solves the H–L pairing challenge by mutating only four amino acid residues in the Fab arm interface. As such, these “BIClonals” may be useful in many biotechnological and therapeutic applications. Still, perhaps the most important take-home message is that, currently there are more than 120 bsAb formats, >30 bsAbs representing many different designs are undergoing clinical evaluation, suggesting they sufficiently satisfied developability requirements [5,55,56]. Obviously, it will be naïve to claim a superior solution for all bispecific IgGs that suits all antibody sequences. We suggest that while converting mAbs to bsAbs, the format should be carefully considered and several options should be evaluated to select the best solution fitting for that particular antibody.

## 5. Patents

The “BIClonals” technology described here is covered by patents US 9,624,291 B2: “Bi- and monospecific, asymmetric antibodies and methods of generating the same” and Eu patent EP 2 686 348 B1: Bi- and monospecific, asymmetric antibodies and methods of generating the same. The priority date is 15 March 2012.

## Figures and Tables

**Table 1:** The selected positions for introducing cysteine mutations in domains C_H1_ and C_L_. The Cα-Cα distance between the residues of each pair is indicated.

Molecule Name	Residue in C_H1_	Residue in C_L_	Cα-Cα Distance (Å)
A	F174	S162	6.4
B	F174	T164	5.6
C	L124	F118	6
D	F122	S121	6.8
E	F122	E123	7
F	F122	Q124	6.7
G	S130	F118	6.7

**Table 2:** Summary of the EC_50_ values of “Arginine mutants” family as were extracted from ELISA binding curves to streptavidin.

Molecule Name	Heavy Chain	Light Chain	EC_50_ (nM)
α SA WT	α SA WT	α SA WT	0.15
1	α SA WT	C214A	0.05
2	C222A	α SA WT	0.15
3	α SA WT	C214R	0.09
4	C222R	α SA WT	0.1
5	C222R	C214R	0.2
6	C222A	C214A	0.05
7	C222R	C214A	0.25
8	C222A	C214R	0.08

**Table 3:** Tm values (°C) of the bsAbs vs. their parental antibodies as determined by NanoDSF. The bsAbs all have a KIH Fc and the mutations changing the cysteines of the naturally-occurring H–L disulfide bond of the engineered Fab arm mutated to Alanine in the H chain and deleted in the Kappa light chain. In the YY mutants they are changed to tyrosine.

Molecule Name and Mutations	Tm1 (°C)	Tm2 (°C)
*H* (Heavy chain C44/Light chain C100)	67.0	76.8
*B* (Heavy chain C174/Light chain C164)	67.3	77.1
*E* (Heavy chain C122/Light chain C123)	67.7	77.5
*BYY* (B with Heavy chain Y222/Light chain Y214)	67.6	75.2
*EYY* (E with Heavy chain Y222/Light chain Y214)	68.0	76.1
T427 WT	67.0	81.3
αSA WT	70.2	78.2

**Table 4:** Calculated production yields of all bsAbs produced in Expi293™ mammalian cells.

Molecule Name	Yield (µg/30 mL Transfection)	Total Calculated Yield (mg/L)
*H*	230	7.7
*B*	525	17.5
*E*	360	12
*BYY*	610	20.3
*EYY*	215	7.2
T427 WT	2.6 mg *	86
αSA WT	10 mg *	330

* The yields for the WT mAbs are following single-step MabSelect purification. The BsAbs were purified by a two-step KappaSelect followed by LambdaSelect affinity chromatography.

**Table 5:** Binding kinetics of the bsAbs and their parental mAbs to CD30 and SA antigens, as determined by SPR. Surface plasmon resonance binding affinities collected from a Biacore T200, of the investigated bsAbs and their parental antibodies to CD30 and SA.

Antibody	Binding to CD30			Binding to SA		
	K*a* (1/Ms)	K*d* (1/s)	K_D_ (Nm)	K*a* (M-1s-1)	K*d* (1/s)	K_D_ (nM)
*H*	2.04 × 10^5^	2.97 × 10^−3^	14.6	5.26 × 10^4^	1.93 × 10^−3^	36.6
*B*	1.67 × 10^5^	1.99 × 10^−3^	11.9	1.29 × 10^5^	2.35 × 10^−3^	18.2
*E*	1.34 × 10^5^	1.80 × 10^−3^	13.5	1.28 × 10^5^	2.48 × 10^−3^	19.3
*BYY*	2.00 × 10^5^	2.29 × 10^−3^	11.5	4.30 × 10^4^	2.65 × 10^−3^	61.7
*EYY*	1.21 × 10^5^	1.94 × 10^−3^	16	2.20 × 10^4^	3.18 × 10^−3^	145
T427 WT	4.69 × 10^5^	4.22 × 10^−4^	0.899	-	-	-
αSA WT	-	-	-	3.68 × 10^5^	1.41 × 10^−4^	0.384

* The constants are empirical and not mechanistic.

## Supplementary Materials

The following are available online at http://www.mdpi.com/2073-4468/7/3/27/s1.
