# Supplementary material for: Design Principles for Bispecific IgGs, Opportunities and Pitfalls of Artificial Disulfide Bonds
Source: Antibodies (Basel). 2018 Jul 28;7(3):27. doi: 10.3390/antib7030027 (PMC6640675; doi:10.3390/antib7030027)
Supplement: Supplementary file 1 [file antibodies-07-00027-s001.pdf]

antibodies

MDPI

Article

Design Principles for Bispecific IgGs, Opportunities and Pitfalls of Artificial Disulfide Bonds

Lilach Vaks [1], Dana Litvak-Greenfeld [1], Stav Dror [1], Galia Matatov [1], Limor Nahary [1], Shiran Shapira [2], Rahely Hakim [3], Iris Alroy [3] and Itai Benhar [1,*]

[1] School of Molecular Cell Biology and Biotechnology, The George S. Wise Faculty of Life Sciences, Tel Aviv University, Tel Aviv, Israel; linais07@gmail.com (L.V.); dana.litvak@gmail.com (D.L.-G.); stavdror88@gmail.com (S.D.); galiamatatov@mail.tau.ac.il (G.M.); naharyl@yahoo.com (L.N.)

[2] Integrated Cancer Prevention Center, Tel Aviv Sourasky Medical Center, Sackler Faculty of Medicine, Tel Aviv University, Tel Aviv, Israel; shiranshapira@gmail.com

[3] FusiMab, Ltd., 14 Shenkar St. POB 4093 Herzelia, Israel; rahely.hakim@gmail.com (R.H.); iris.alroy@animabiotech.com (I.A.)

* Correspondence: benhar@tauex.tau.ac.il; Tel.: +972-3-640-7511

Supplementary Materials:

Method S1: Production of antibodies in an *E. coli* expression-refolding system. Some of the antibodies that were produced during the study were made by bacterial expression using the "Inclonals" method for production of full-length IgGs in *E. coli* bacteria [1]. The individual heavy and light chains were expressed in *E. coli* BL21 (DE3) pUBS500 cells that were transformed with pHAK-Light chain and pHAK-Heavy chain-based vectors. Cells were grown in 0.5 liter of SB medium, induced for protein expression and inclusion bodies were purified essentially as described [1]. The purified inclusion bodies were solubilized in 6 M guanidinium hydrochloride containing buffer at a protein concentration of 10 mg/ml, mixed at a heavy to light chain molar ratio of 1:2. For production of bsAbs, the heavy chains (and the light chains) of the different antibodies were mixed at a ratio of 1:1, thus requiring 4 chains (2 heavy and 2 light). The inclusion bodies mix was then reduced with 10 mg/ml DTE for 2 h and refolded by ×100 dilution into refolding solution containing redox shuffling and aggregation-preventing additives for 36 h at 10ºC. Following refolding, the protein solution was concentrated and its pH was reduced to pH 7.2 by diafiltration using MasterFlex® L/S diafitration device (Cole Palmer, USA) with a 100 kDa cutoff cassette and 50 mM Tris(HCl), pH 7.0, 0.3 M arginine monohydrochloride (50 gr/liter) diafiltration buffer. The refolded

protein was then filtered using a 0.45 µm filter and separated from contaminating bacterial proteins, excess light chains and improperly folded proteins by protein-A (or MabSelect) affinity chromatography. IgGs were eluted from the column using an acidic citric acid buffer, neutralized with 1 M Tris (HCl) pH 8.5 and dialyzed against 1000 volumes of PBS. In some cases, the IgG was further purified by preparative SEC using a Superdex 200 column (GE Healthcare) that was developed using PBS as the running buffer at 0.5 ml/min. Purified IgGs were stored at -80ºC.

Method S2: Stable antibody expression in CHO cells. Heavy and light chain DNA sequences were cloned into GPEx® vectors (Catalent Inc, USA), a pseudo typed, high-titer retroviral vector which generated stable transduced mammalian cells. The cloned retroviral vectors (4 different vectors, each encoding a single light or a single heavy chain) were used to transduce CHO cells, and genomic copy number of light and heavy chain were determined (light-to-heavy ratio of ~1.5:1). Cells were grown in suspension in protein free medium (PF-CHO) and antibody containing cell culture supernatants were harvested after 7 d of cultivation in shake flasks by centrifugation at 14,000 × g for 30 min and clarified by filtration through a Meissner Protec GF filter followed by 0.2 µm filter. The antibody was purified according to standard protocols by Protein-A affinity chromatography (MabSelect, GE Healthcare), followed by elution of antibodies at pH 3.0 and immediate neutralization of the sample.

Method S3: SDS-PAGE electrophoresis. Polyacrylamide gel electrophoresis of proteins was performed according to Laemmli [2]. 1/5 volume of 5×sample buffer was added to the protein samples followed by boiling for 5 min prior to the loading onto the gel. 7.5%, 10% and 12% mini-gels were run at 120V. Gels were stained with GelCode blue® solution for 15 min and washed in water. The protein band density was analyzed by ImageMaster 1D scanning laser densitometer (Pharmacia, Sweden). Gels that were stained were loaded with 20 µg of protein per lane for non-purified fraction or 3-5 µg for purified proteins. Gels that were further processed by immunoblotting were loaded with 1/10 of these quantities.

Method S4: Western Blot (immunoblot) analysis. Proteins resolved by SDS-PAGE were electro-transferred onto the nitrocellulose membrane and blocked for at least 1 h with PBS containing 5% non-fat milk powder at room temperature with slow agitation. The membrane was washed with PBS followed by incubation HRP conjugated goat-anti-human (H+L) secondary antibodies (Jackson Immunoresearch Laboratories, USA). After three washes with PBST (PBS containing 0.05% Tween 20) and one wash with PBS the nitrocellulose filter was developed with the SuperSignal West Pico Chemiluminescent Substrate (Thermo Scientific) as recommended by the supplier.

Method S5: ELISA. The antigen binding by mono- and bi-specific IgGs was determined as follows: 96-well ELISA plates were coated with 5 µg/ml of pure protein antigens in PBS 50 µl/well, 14 h at 4°C and blocked with 3% skim milk in PBS for 1 h at 37°C. All subsequent steps were carried out at room temperature (about 25°C). Purified antibodies (or cell conditioned media) were applied onto the plates in a three-(or four) fold dilution series in PBST for 1 h incubation and washed with PBST for three times. Following the 1 h incubation with an HRP-conjugated secondary antibody (1:5000 dilution in PBST, 100 µl/well) the plates were washed in PBST and developed using 50 µl/well of chromogenic HRP substrate TMB (Dako). Color development was terminated with 50 µl/well of 1M H_2SO_4. The plates were read at 450 nm. Antibodies were tested in triplicate wells and the results are presented as the mean ± standard deviation (STDEV) of the values.

Method S6: Surface plasmon resonance. Experiments for evaluation of antigen binding kinetics and determination of affinity constant of the antibodies were performed using the Biacore T200 instrument [3]. The two antigens, CD30 and SA, to which the binding of the antibodies was evaluated, were diluted in 150mM sodium acetate buffer (pH 4.6) and each of them was immobilized to the surface of one of the channels of a Series S CM5 Sensor Chip using standard amine-coupling chemistry (NHS-EDC) to a surface density of ~1300 and ~450 response units (RU), respectively. After capturing the antigens to the surface, each

of the bsAbs and their parental antibodies were injected at increasing concentrations (1, 3, 4, 5, 7. 10, 20, 30, 50 and 70 nM) at a flow rate of 10 µl/min for 120 sec. Monovalent T427 was injected as well over the SA-immobilized surface as negative control (data not shown). The dilutions of the antibodies were performed in HBS-EP+ buffer – 10 mM HEPES-buffered saline (pH 7.4) containing 150 mM NaCl, 3 mM EDTA, and 0.05% Surfactant P20 (Tween 20). The antibodies were allowed to dissociate for 240 sec and after another interval of 60 sec after each sample, the surface was regenerated with NaOH for 60 sec. The dilution series sensograms generated for each antibody were fit to a 1:1 binding model to determine the binding (K*a*) and dissociation (K*d*) rate constants and the equilibrium dissociation constant (K_D). The analysis was conducted using the BIAevaluation software [3]. Bispecific binding ability was demonstrated by conducting the "sandwich SPR" assay in which VEGF was were immobilized onto the surface of a CM5 sensor chip using standard amine-coupling chemistry to a surface density of about 1200 RU. The bsAb was injected for 5 min followed by 1-min injection of hAng-2 (R&D Systems) (both molecules at a concentration of 10 µg/mL). Data for this experiment were globally fit using the BIAevaluation 3.2 software package.

Method S7: Thermal stability assay. Thermal unfolding experiments are highly appreciated methods to quantify protein stability. The thermal stability of a given protein is typically described by the thermal unfolding transition midpoint Tm (°C), at which half of the protein population is unfolded. In this study we used NanoDSF assay for determination of the Tm values of each of the investigated antibodies. NanoDSF (DSF=Differential Scanning Fluorimetry) measurements were performed using the Prometheus NT.48 instrument by NanoTemper Technologies GmbH [4]. The basis of the dye-free NanoDSF method is the detection of even minute changes in the emission properties of the amino acid tryptophan of the examined protein upon unfolding. This is done by a dual-UV technology that monitors intrinsic tryptophan fluorescence at the emission wavelengths of 330 and 350 nm. When located in the hydrophobic core of proteins, tryptophan is shielded from the surrounding aqueous solvent. Upon unfolding however, tryptophan is exposed, which alters its photo-physical properties. To generate an unfolding curve, the ratio of the fluorescence

intensities (F350 nm/F330 nm), is plotted against temperature. To remove aggregates, each antibody was centrifuged at 14,000 rpm, for 10 min, at 4° C before the NanoDSF measurement. The assay was performed by filling small capillaries with 10 µl sample for each antibody. Each measurement was performed in duplicates. The capillaries were then loaded into the Prometheus NT.48 Instrument where the antibodies were subjected to a temperature ramp of 1°C/min, from 20 to 95°C. Tryptophan fluorescence was constantly monitored at 350 and 330 nm. The Tm values, corresponding to the inflection points of the unfolding curve, were determined automatically by the PR.Control software via the first derivative of the curve.

Method S8: MTT cell viability assay. 1×10^4 cells/well (100 µl) were seeded in 96-well plates DMEM medium (supplemented with 10% FCS, 2% L-Glutamine and 1% penicillin-streptomycin-nystatin antibiotics) for 24 h. Then, the medium was replaced with 100 µl/well of varying concentrations of evaluated proteins diluted in the same medium and the plate were further incubated 72 h at 37°C. The assay ended with the vacuum aspiration of the growth media and the addition of 100 µl/well of 1 mg/ml thiazolyl blue tetrazoliam bromide (MTT) for 1 h at 37°C. The MTT-formazan crystals were dissolved by the addition of 100 µl/well of the MTT extraction buffer and overnight incubation at 37°C. Cell viability was calculated from the absorbance values read at 570 nm in the Synergy (Biotek) plate reader. The results acquired from at least three independent experiments performed in triplicates were expressed as percentage of living cells in respect to the untreated controls that were processed simultaneously using the following equation:

$$\% \text{ Viability} = (A_{570nm} \text{ of treated sample}/A_{570nm} \text{ of untreated sample}) \times 100.$$

Method S9: Determination of mouse blood pharmacokinetics of BIC101 and BIC201. BIC 101 is an anti VEGF – anti Ang2 bsAb and BIC 2010 produced by refolding while BIC201 is the same bsAb produced in CHO cells. Experiments were performed in female CD-1 mice using a single i.v. bolus administration of 5 mg/kg BIC101 or 10 mg/kg BIC201.

Concentrations of the human antibodies in mice serum were determined by ELISA using Ang2 as an antigen.

Method S10: Colo205 Tumor xenograft model.

Colo205 cells (ATCC) (1×10^6 cells suspended in 200 μl BD Matrigel Matrix (BD Biosciences) /PBS (1:1)) were injected s.c. in the right flanks of 9-wk-old female athymic nude mice (Crl:NU(Ncr)-Foxn1nu, Charles River). Tumor growth was quantified weekly by caliper measurements. Tumor volume was calculated by measuring with a caliper the largest diameter (A) and its perpendicular (B) according to the formula, $0.5 \times A \times B^2$. Mice were treated i.p. with 10 mg/kg bevacizumab, with 10 mg/kg BIC201, or with 20 mg/kg BIC201. Rituximab, a chimeric anti-CD20 antibody, was used as an isotype-matched negative control at 10 mg/kg (i.p.) Mice were treated once weekly for a period of 6 weeks, and tumor volume was monitored for a total of 50 days.

Method S11: Production of biotinylated AVITAG-PE38 fusion protein. This biotinylated toxin fragment was produced as a model effector molecule to be delivered to $CD30^+$ target cells by a bsAb with the anti CD30 antibody T427 as one arm and anti-streptavidin (αSA) as the second arm. A plasmid named pET22-AVITAG-PE38 was constructed to allow the expression of soluble Pseudomonas exotoxin A fragment (PE38) [5] to which the 15-AA length AVITAG (BirA tag, GLNDIFEAQKIEWHE that provides for efficient site-specific biotinylation of the fused protein by the *E. coli* BirA enzyme [6]) was fused at the N-terminus of PE38. The protein was secreted to the periplasm of BL-21 (DE3) *E. coli* cells and purified from periplasmic fractions essentially as described for other secreted PE derivatives [5]. AVITAG-PE38 was used as a substrate for *in vitro* biotinylation by MBP-BirA biotinylation enzyme. AVITAG-PE38 was diluted to final concentration of 1 mg/ml in BirA reaction buffer (10 mM Tris-HCl pH 8, 7.5 mM MgCl2, 5 mM NaCl) with 2 mM ATP and 10 mM D-biotin. MBP-BirA enzyme at a final concentration of 1 μg/ml and the reaction was left at 25˚C overnight.

Method S12: Evaluation of the cytotoxic activity of the biotinylated AVITAG-PE38 fusion protein. An antibody-PE38 toxin complex was formed by incubation of the biotinylated AVITAG-PE38 with streptavidin at 1:1 molar ratio in PBS, at room temperature for 1 h. Next, 2-folds molar excess of biotinylated bsAb was added for 1 h incubation. Varying concentrations of the resulted complex were added in triplicate wells to human epidermoid carcinoma A431/CD30 cells that were previously seeded in 96-well plates at a density of 1×10^4 cells/well in DMEM medium supplemented with 10% FCS. Cells were incubated for 48 h at 37°C in 5% CO_2. After treatment, the media was replaced by immunotoxin-free media (125 µl per well) and an MTT assay was carried out as described in the **Methods** section.

Result S1. The light chain of the anti SA IgG is crucial for SA binding. The anti SA antibody we used is a human antibody that was isolated as a scFv with a lambda light chain from a phage library and converted to a human IgG1 for this study. To evaluate the contribution of the heavy and light chains of the anti SA antibody, two refolded IgGs were prepared. One was the WT anti SA IgG and the second composed of the heavy chain of anti SA refolded with the light chain of the anti CD30 antibody T427. The refolded and purified antibodies were evaluated for SA binding by ELISA. As shown in **Fig. S1**, the SA binding by the IgG with the light chain of T427 was severely compromised. These results suggest that in this anti SA antibody, a significant part of the binding interactions with the antigen are provided by the light chain.

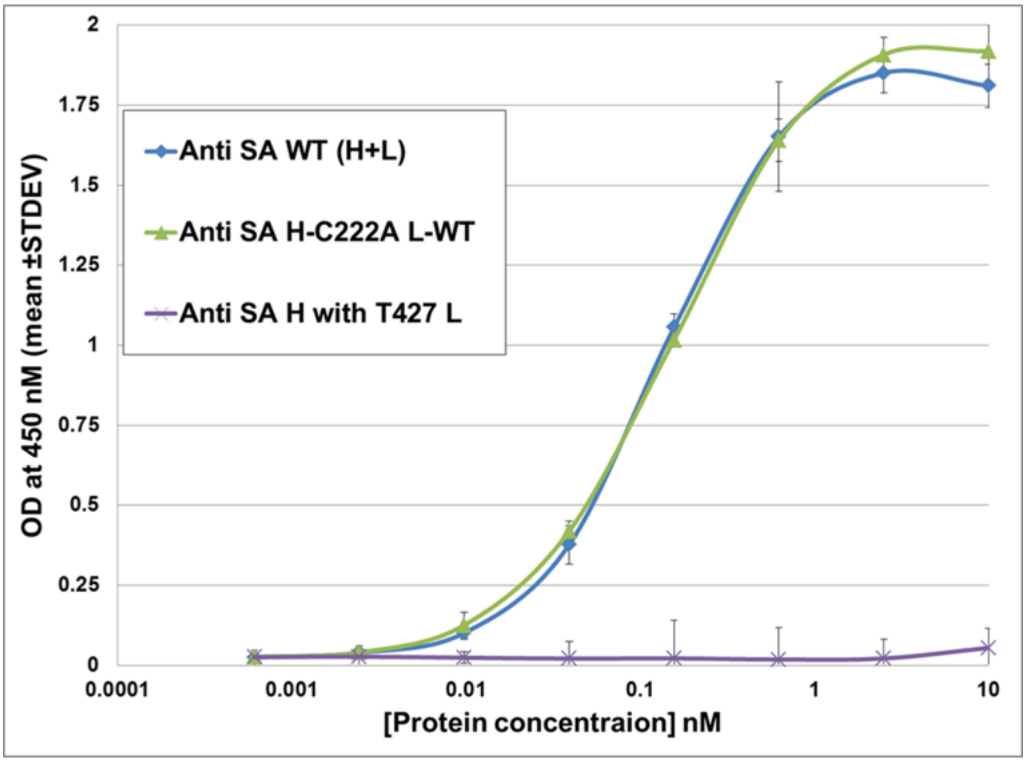

Figure S1: The light chain of the anti SA IgG is crucial for SA binding. Three refolded IgGs were evaluated for binging to plate-immobilized SA by ELISA. Binding was detected using an HRP-conjugated goat anti human IgG (H+L).

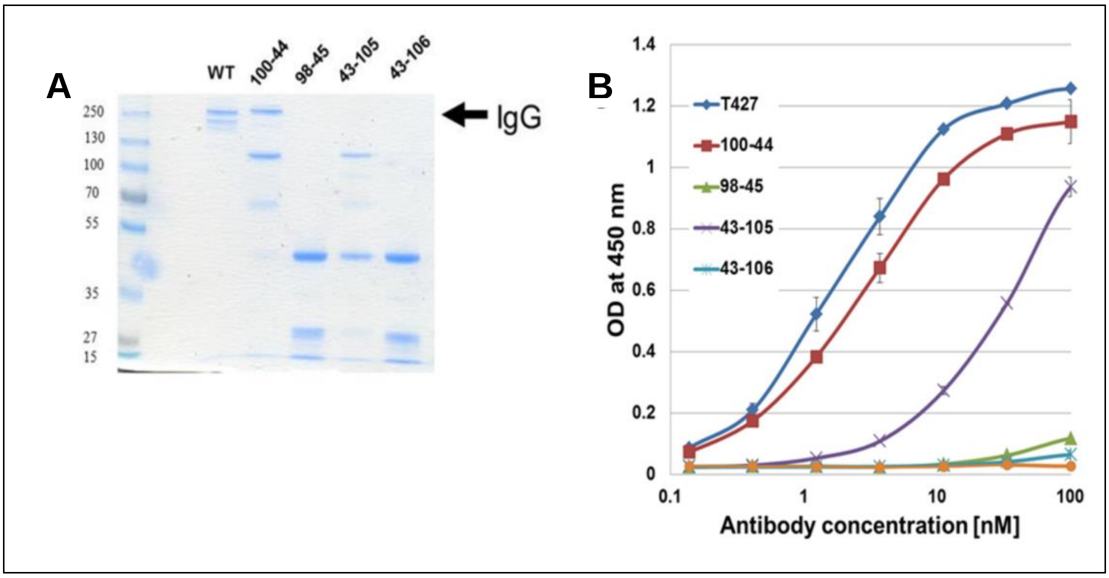

Figure S2. Evaluation of different positions of artificial disulfide bonds for stabilizing the Fab arm interface of "disulfide-stabilized IgGs". T427 (κ) IgG was used as the model antibody. **(A)** SDS-PAGE analysis (10% acrylamide under non-reducing conditions) of T427 IgG variants. **(B)** CD30 binding of T427 IgG variants evaluated by ELISA.

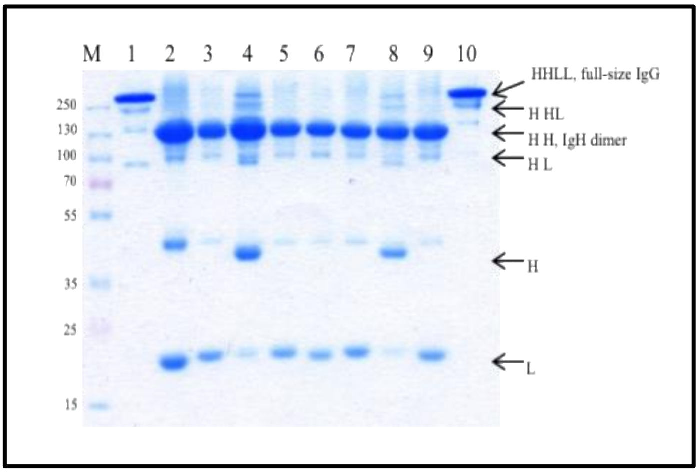

Figure S3. Analysis of “disulfide-stabilized IgG” antibodies of "arginine mutants" of anti SA by SDS/PAGE. M) protein marker 1. αSA WT (Heavy chain αSA WT+Light chain αSA WT) 2. Heavy chain αSA WT+Light chain αSA C214A; 3. Heavy chain αSA WT+Light chain αSA C214R; 4. Heavy chain αSA C222R+ Light chain αSA WT; 5. Heavy chain αSA C222R+ Light chain αSA C214R; 6. Heavy chain αSA C222R+Light chain αSA C214A; 7. Heavy chain αSA C222A+ Light chain αSA C214R; 8. Heavy chain αSA C222A+ Light chain αSA WT; 9. Heavy chain αSA C222A+ Light chain αSA C214A. 10. Erbitux (used as a full-size IgG protein marker). 5 µg of each purified antibody were separated on a 10% SDS-PAGE under non-reducing and conditions and visualized using GelCode Blue™ Staining.

References

1. Hakim, R.; Benhar, I. "Inclonals": IgGs and IgG-enzyme fusion proteins produced in an *E. coli* expression-refolding system. *mAbs* **2009**, *1, 281-287*.
2. Laemmli, U.K. Cleavage of structural proteins during the assembly of the head of bacteriophage T4. *Nature* **1970**, *227, 680-685*.
3. GE Healthcare. *Biacore™ Assay Handbook*; General Electric Company: Boston, MA, USA, 2012.
4. 20. Senisterra, G.; Chau, I.; Vedadi, M. Thermal denaturation assays in chemical biology. *Assay Drug Dev. Technol.* **2012**, *10, 128–136*.
5. Benhar, I.; Wang, Q.C.; FitzGerald, D.; Pastan, I. Pseudomonas exotoxin a mutants. Replacement of surface-exposed residues in domain III with cysteine residues that can be modified with polyethylene glycol in a site-specific manner. *The Journal of biological chemistry* **1994**, *269, 13398-13404*.
6. Beckett, D.; Kovaleva, E.; Schatz, P.J. A minimal peptide substrate in biotin holoenzyme synthetase-catalyzed biotinylation. *Protein science : a publication of the Protein Society* **1999**, *8, 921-929*. doi: 10.1110/ps.8.4.921.
